# Somatic mouse models of gastric cancer reveal genotype-specific features of metastatic disease

**DOI:** 10.1038/s43018-023-00686-w

**Published:** 2024-01-04

**Authors:** Josef Leibold, Kaloyan M. Tsanov, Corina Amor, Yu-Jui Ho, Francisco J. Sánchez-Rivera, Judith Feucht, Timour Baslan, Hsuan-An Chen, Sha Tian, Janelle Simon, Alexandra Wuest, John E. Wilkinson, Scott W. Lowe

**Affiliations:** 1 Cancer Biology and Genetics Program, Sloan Kettering Institute, Memorial Sloan Kettering Cancer Center, New York, NY USA; 2 Department of Medical Oncology and Pneumology, University Hospital Tuebingen, Tuebingen, Germany; 3 iFIT Cluster of Excellence EXC 2180 ‘Image-Guided and Functionally Instructed Tumor Therapies’, University of Tuebingen, Tuebingen, Germany; 4 Cold Spring Harbor Laboratory, Cold Spring Harbor, New York, NY USA; 5 David H. Koch Institute for Integrative Cancer Research, Massachusetts Institute of Technology, Cambridge, MA USA; 6 Department of Biology, Massachusetts Institute of Technology, Cambridge, MA USA; 7 Department I–General Paediatrics, Haematology/Oncology, University Children’s Hospital Tuebingen, Tuebingen, Germany; 8 Department of Biomedical Sciences, School of Veterinary Medicine, The University of Pennsylvania, Philadelphia, PA USA; 9 Department of Pathology, University of Michigan School of Medicine, Ann Arbor, MI USA; 10 Howard Hughes Medical Institute, Memorial Sloan Kettering Cancer Center, New York, NY USA

**Keywords:** Cancer models, Cancer genetics, Tumour immunology, Cancer

## Abstract

Metastatic gastric carcinoma is a highly lethal cancer that responds poorly to conventional and molecularly targeted therapies. Despite its clinical relevance, the mechanisms underlying the behavior and therapeutic response of this disease are poorly understood owing, in part, to a paucity of tractable models. Here we developed methods to somatically introduce different oncogenic lesions directly into the murine gastric epithelium. Genotypic configurations observed in patients produced metastatic gastric cancers that recapitulated the histological, molecular and clinical features of all nonviral molecular subtypes of the human disease. Applying this platform to both wild-type and immunodeficient mice revealed previously unappreciated links between the genotype, organotropism and immune surveillance of metastatic cells, which produced distinct patterns of metastasis that were mirrored in patients. Our results establish a highly portable platform for generating autochthonous cancer models with flexible genotypes and host backgrounds, which can unravel mechanisms of gastric tumorigenesis or test new therapeutic concepts.

## Abstract (web summary)

Leibold and colleagues develop a method to somatically introduce oncogenic lesions into the stomach epithelium, which recapitulates the histological, molecular and clinical features of human gastric cancer.

## Main

Gastric cancer is the fourth leading cause of cancer-associated deaths and the fifth most commonly diagnosed cancer worldwide^1^. While localized disease can be cured in about half of patients, effective treatment strategies are currently lacking for advanced and especially metastatic disease, resulting in abysmal survival rates^2–4^.

Genome sequencing studies have classified gastric cancer into four major molecular subtypes. One subtype is defined by Epstein–Barr Virus infection and, because of its viral etiology, we do not consider it further here. The remaining subtypes are defined by (1) chromosomal instability (CIN), (2) genomic stability (GS) and (3) microsatellite instability (MSI)^5–7^. The CIN subtype, which is the most common, typically harbors *TP53* mutations and a high frequency of recurrent copy-number alterations (CNAs)^8^. GS tumors display far fewer chromosomal aberrations and are devoid of *TP53* mutations, instead frequently harboring mutations that inactivate *CDH1* or activate WNT signaling. CIN and GS tumors also differ in their histopathology; CIN tumors show prominent features of intestinal differentiation, whereas GS tumors show diffuse histological features^9^. The MSI subtype is defined by the presence of MSI and mutations in mismatch repair genes such as *MLH1* or *MSH2*. Presumably due to their increased mutational load and potential for neoantigen production, MSI tumors elicit a T cell-dominated immune response^10,11^ and frequently respond to immune-checkpoint blockade^12–14^. Notably, mutational gains and amplifications of the *MYC* gene, which can be found in all gastric cancer subtypes, are associated with early progression of intestinal metaplasia to gastric cancer^5,8^.

Genetically engineered mouse models (GEMMs) are valuable for understanding genotype–phenotype relationships and for evaluating new therapeutic concepts in a range of tumor types; however, due to the need to intercross various germline strains, traditional GEMMs are time- and resource-consuming, making it difficult to model and interrogate the spectrum of tumor genotypes that exist in patients or to conduct large-scale preclinical studies^15–17^. Likewise, interrogating the genetics of tumor–host interactions is cumbersome, requiring a prohibitive number of intercrosses to produce a genetically defined cancer in an altered host strain. For gastric cancer, existing GEMMs only model some molecular subtypes on a single host background and, in contrast to patients, rarely progress to metastatic disease^18^. Therefore, new models that capture the genetic diversity and metastatic progression of human gastric cancer and enable facile changes in the host could transform the study of this disease.

We and others have devised methods to somatically introduce cancer-predisposing lesions or other genetic elements into murine tissues using electroporation, thereby producing electroporation-based GEMMs (EPO-GEMMs)^19–22^. In this approach, transposon-based vectors encoding complementary DNAs or CRISPR-Cas9 constructs targeting endogenous genes are introduced into the tissue via survival surgery through a brief electric pulse, whereby they are taken up by a subset of cells. If a particular lesion or combination of lesions provides a selective advantage, focal tumors arise at the electroporation site. Herein, we developed surgical methods and electroporation conditions suitable for engineering mice with gastric tumors harboring a range of cancer genotypes and show that the resulting platform can faithfully model the three major nonviral subtypes of the human disease. Furthermore, we illustrate the power of combining this approach with mice of different genetic backgrounds to explore tumor–host interactions relevant to metastatic spread. The portability, flexibility and speed of these gastric EPO-GEMMs creates new possibilities for exploring how gastric cancers evolve, spread and respond to therapy in the complex in vivo environment.

## Results

### EPO-GEMMs of CIN and GS gastric cancer

**Fig. 1:**
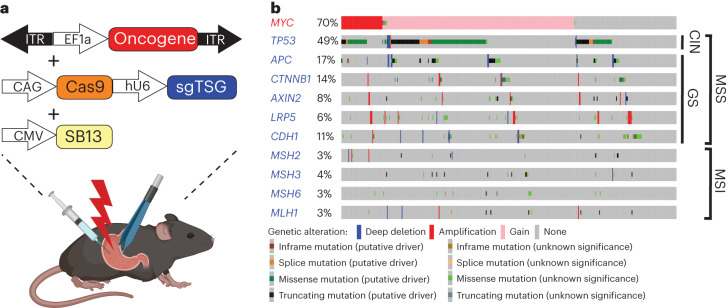
Modeling molecular subtypes of gastric cancer in mice by a somatic tissue engineering approach. **a**, Schematic of the EPO-GEMMs of gastric cancer. A transposon vector harboring an oncogene in combination with a Sleeping Beauty transposase (SB13) and a CRISPR-Cas9 vector targeting tumor-suppressor genes are delivered into the stomach by direct in vivo electroporation. **b**, MSK-IMPACT oncoprint displaying the genomic status of recurrent oncogenes and tumor-suppressor genes in patients with gastric cancer. Copy-number gains are shown for *MYC*. Associated molecular subtypes (per TCGA^8^) are shown on the right. Source data

**Extended Data Fig. 1:**
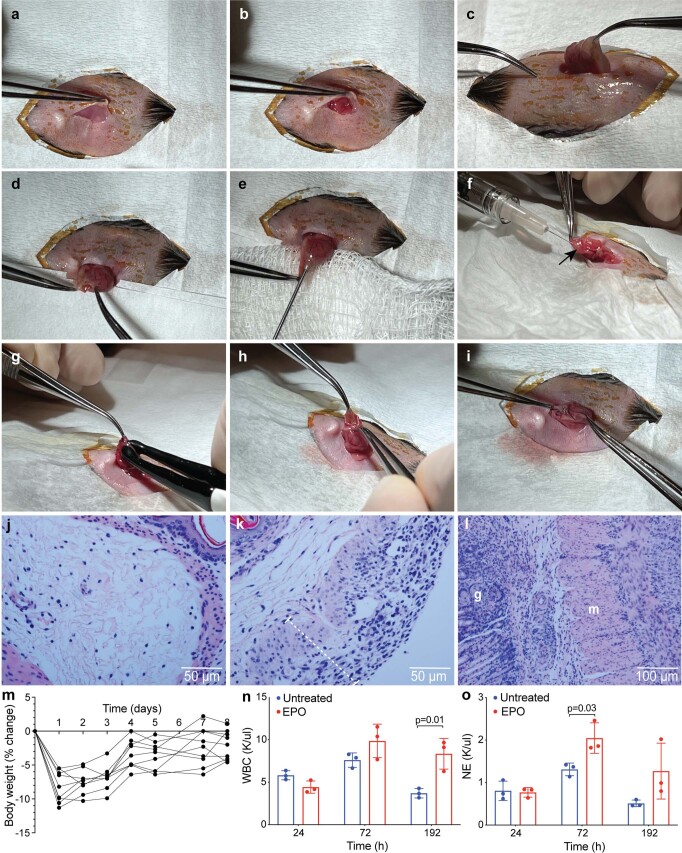
Description of gastric EPO-GEMM procedure and analysis of body weight and inflammation post-electroporation. (A-I) Step-by-step illustration of the EPO-GEMM procedure: (**a, b**) After hair removal, scrubbing of the surgical site with povidone-iodine, and rinsing with 70% alcohol, incisions of the skin (A) and the peritoneum (B) of about 7 mm each are made in the epigastric region. (**c**) The stomach is localized in the peritoneal cavity and mobilized to the exterior using a curved forceps. The right forceps in the image holds the forestomach. (**d**) To open the stomach, a 4-mm incision is made in the forestomach. (**e**) The stomach is rinsed with prewarmed saline using a flexible oral gavage needle to remove any remaining food. (**f**) The epithelial part of the stomach is mobilized to the exterior through the incision in the forestomach using a curved forceps, and the plasmid mix is injected into the epithelial layer of the corpus region using a 30G needle. A small liquid filled bubble forms around the injection area (arrow). (**g**) The Nepagene tweezer electrodes are loosely placed around the plasmid-containing bubble and electric pulses are applied by the Nepagene electroporator. (**h-i**) The stomach is reversed, and the forestomach and all the layers are sutured at once in a continuous seam using absorbable sutures (Ethicon 5-0). Afterwards, the peritoneal cavity is closed by single knot sutures and the incision in the skin is closed by surgical staples. (**j–l**) Extent of inflammation after electroporation with vehicle only. (J) Edema with limited influx of neutrophils at the electroporation site seen 24 h after EPO, consistent with early signs of local inflammation. Representative image of n = 3 mice. (K) Influx of neutrophils in the mucosal area (dashed line) at the electroporation site seen 72 h after EPO, consistent with a developing inflammatory response. Representative image of n = 3 mice. (L) Acute inflammatory response characterized by influx of neutrophils in the glandular (g) and muscle (m) layers of the stomach at the electroporation site, seen 192 h after EPO. Representative image of n = 3 mice. (M) Changes in animal body weight over 8 days following the EPO procedure. Individual lines represent independent mice (n = 9 mice). (**n–o**) Levels of white blood cells (N) and neutrophils (O) in the peripheral blood of mice at the indicated time-points following electroporation with vehicle only. Untreated mice that were not subjected to surgery or EPO served as controls (n = 3 independent mice per group). Statistical analysis by unpaired two-sided t-tests. Data are presented as mean values +/− s.e.m. Source data

To generate gastric cancer EPO-GEMMs, survival surgery was coupled with direct tissue electroporation to deliver genetic elements to the murine stomach epithelium (details on the procedure are in Extended Data Fig. 1a–i and Methods). The surgery was well tolerated, with over 97% of the animals surviving the procedure. It resulted in temporary weight loss and signs of local and systemic inflammation, which began to resolve within a week (Extended Data Fig. 1j–o). The genetic elements in the gastric cancer EPO-GEMMs consisted of a transposase–transposon vector pair, used to express a defined oncogene, and a plasmid coexpressing Cas9 with a single-guide RNA (sgRNA), used to knock out a tumor-suppressor gene of interest (Fig. 1a). Because *MYC* is frequently (~70%) gained or amplified across gastric cancers^5,8^, we used a transposon vector containing human *MYC* cDNA as the universal oncogene and adapted sgRNAs to target different tumor-suppressor genes in accordance with their mutation in distinct subtypes of gastric cancer (Fig. 1b).

**Fig. 2:**
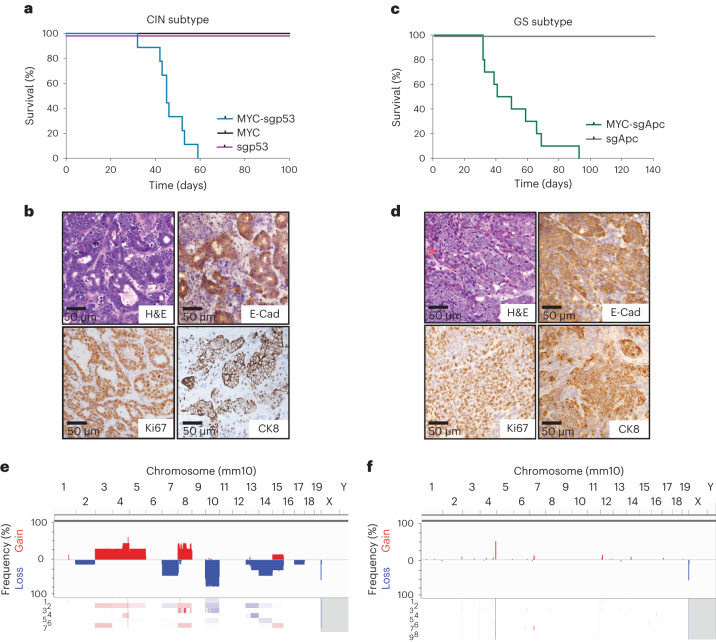
CIN and GS gastric cancer EPO-GEMMs recapitulate hallmark histological and molecular features of the corresponding human subtypes. **a**, Kaplan–Meier survival curves of C57BL/6 mice electroporated with a *MYC* transposon vector and a Sleeping Beauty transposase (*MYC*; black, *n* = 4 mice); a CRISPR-Cas9 vector targeting *p53* (sgp53; purple, *n* = 4 mice) or the combination of all vectors (MYC-sgp53; blue, *n* = 9 mice). **b**, H&E and immunohistochemical staining for E-cadherin (E-Cad), Ki67 and cytokeratin 8 (CK8) of a *MYC-p53*^−/−^ gastric EPO-GEMM tumor. Representative of *n* = 9 mice. **c**, Kaplan–Meier survival curves of C57BL/6 mice electroporated with a CRISPR-Cas9 vector targeting *Apc* (sgApc; gray, *n* = 3 mice) or with the combination of Sleeping Beauty, a *MYC* transposon vector and a CRISPR-Cas9 vector targeting *Apc* (MYC-sgApc; green, *n* = 10 mice). **d**, H&E and immunohistochemical staining for E-Cad, Ki67 and CK8 of a *MYC-Apc*^−/−^ gastric EPO-GEMM tumor. Representative of *n* = 10 mice. **e**,**f**, Sparse whole-genome sequencing analysis of CNAs in *MYC-p53*^−/−^ (*n* = 7 independent samples derived from separate mice) (**e**) and *MYC-Apc*^−/−^ (*n* = 9 independent samples derived from separate mice) (**f**) gastric EPO-GEMM tumors. Frequency plots are shown on the top and individual sample tracks are provided on the bottom. Source data

**Extended Data Fig. 2:**
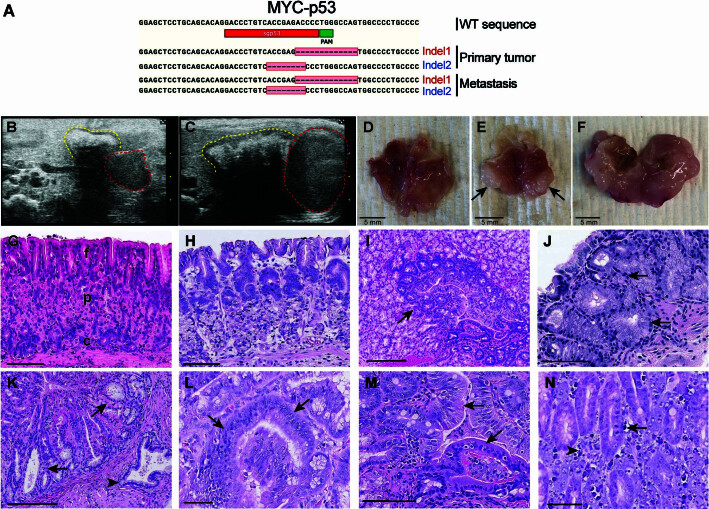
Gastric cancer EPO-GEMMs develop through a stepwise progression of precursor lesions. (**a**) Sanger sequencing results confirming editing of the *Trp53* locus targeted by the indicated CRISPR/Cas9-sgRNA in a MYC-p53 EPO-GEMM gastric tumor. (**b, c**) Representative ultrasound images of developing gastric EPO-GEMM tumors. Dashed yellow line demarcates the stomach; dashed red line indicates the tumors. (**d–f**) Representative gross pathology images of EPO-GEMM stomachs at different stages of tumor development. Tumor formation at the electroporation site is evident in early-stage tumors (E, arrows). (**g–n**) Histological analysis of precursor and early gastric EPO-GEMM lesions. (**g**) Normal murine fundic mucosa (f = foveolar cells; p = parietal cells; c = chief cells) (bar = 500 µm). Representative image of n = 3 mice. (**h**) Atrophy of the fundic mucosa with loss of chief and parietal cells (bar = 250 µm). Representative image of n = 3 mice. (**i**) Hyperplasia in the fundic mucosa (arrow) with increased basophilia of glandular epithelium (bar = 200 µm). Representative image of n = 3 mice. (**j**) Mild to moderate mucinous metaplasia (arrows) in the gastric fundus (bar = 200 µm). Representative image of n = 3 mice. (**k**) Moderate to severe mucinous metaplasia (arrows), hyperplasia, and dysplasia in the gastric fundus. There was also a focus of adenomyosis (arrowhead) (bar = 200 µm). Representative image of n = 3 mice. (**l**) Dysplasia (arrows) of glandular mucosa (bar = 100 µm). Representative image of n = 3 mice. (**m**) Higher magnification of glandular dysplasia (arrows) (bar = 200 µm). Representative image of n = 3 mice. (**n**) Glandular dysplasia, hyperplasia (arrow = mitotic figures), and necrosis (arrowhead) in the gastric fundus (bar = 100 µm). Representative image of n = 3 mice. Source data

To model CIN gastric cancer, we combined the *MYC* transposon–transposase system with a Cas9-sgRNA vector targeting *Trp53* (hereafter referred to as *p53*) to recapitulate a genotype commonly seen in patients^23^ (Fig. 1b). Mice electroporated with all three plasmids consistently developed tumors (96% penetrance) that harbored the predicted disruptions of the *p53* locus (Extended Data Fig. 2a). Median survival was 45 d post-electroporation (Fig. 2a). In contrast, mice electroporated with either *MYC* or Cas9-sgp53 vector alone did not develop tumors within 1 year (Fig. 2a).

**Extended Data Fig. 3:**
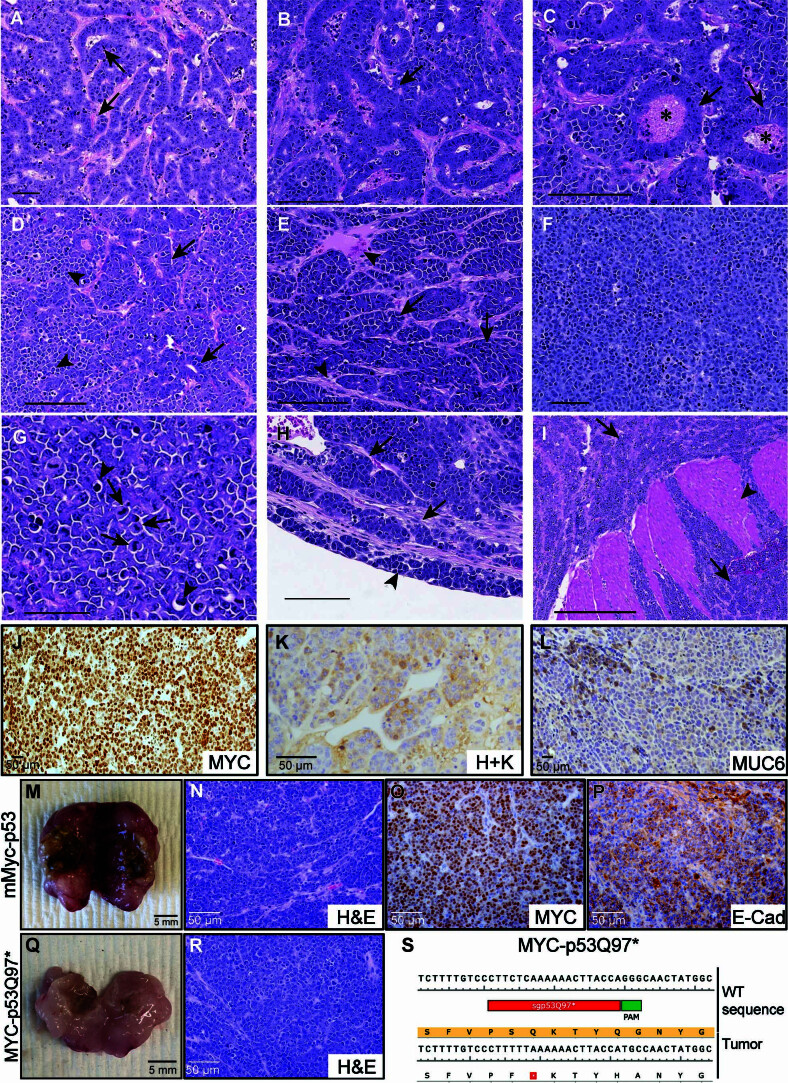
Histopathological analysis of CIN gastric cancer EPO-GEMMs. (**A–I**) Histological characterization of tumor progression in MYC-p53^−/−^ tumors. (A-C) Initially, MYC-p53^−/−^ tumors were moderately well-differentiated adenocarcinomas: (**a**) Areas of tubular gland formation (arrows) (bar = 200 μm). Representative image of n = 3 mice. (**b**) Higher magnification of adenomatous proliferation (arrow) in a typical early tumor. The tumor cells formed single and multiple layers around the lumens (bar = 100 µm). Representative image of n = 3 mice. (**c**) Neoplastic glands (arrows) with cell debris (asterisks) in lumen. Neoplastic glands were lined by tightly adherent pleomorphic columnar and stratified epithelial cells with irregular large nuclei, clumped chromatin, and prominent nucleoli (bar = 100 µm). Representative image of n = 3 mice. (**d**) As MYC-p53^−/−^ tumors progressed, the area of glandular formation (arrows) decreased and blended with larger sheets of tumor cells (arrowheads) typical of a diffuse tumor phenotype (bar = 200 µm). Representative image of n = 3 mice. (**e**) When MYC-p53^−/−^ tumors transitioned from adenomatous to diffuse, the tumors occasionally developed focal areas of micro-lobules (arrows) separated by prominent stroma (arrowheads) (bar = 250 µm). Representative image of n = 3 mice. (**f–i**) Eventually, MYC-p53^−/−^ tumors lost all adenomatous features and became diffuse tumors with solid sheets of neoplastic cells. Representative image of n = 3 mice. (F) These late-stage tumors featured small monomorphic epithelial tumor cells with small basophilic nuclei and scant cytoplasm. The neoplastic cells had a high nuclear/cytoplasm ratio consistent with malignancy (bar = 200 µm). Representative image of n = 3 mice. (G) High magnification of an MYC-p53^−/−^ tumor characterized by sheets of tumor cells with minimal stroma and a diffuse tumor phenotype. There were numerous mitotic figures (arrows) and apoptotic/necrotic cells (arrowheads) (bar = 100 µm). Representative image of n = 3 mice. (H) The tumors were highly invasive and penetrated the submucosa and muscle layers of the stomach (arrows) and spread into the sub-serosa (arrowhead) (bar = 500 µm). Representative image of n = 3 mice. (I) The invasive tumors (arrows) penetrated all layers of gastric muscle (arrowhead) (bar = 200 µm). Representative image of n = 3 mice. (**j-l**) Representative immunohistochemistry staining of a MYC-p53 EPO-GEMM gastric tumor for MYC (J), hydrogen/potassium ATPase (H+K) (K), and MUC6 (L). Representative images of n = 3 mice. (M) Gross pathology image of a mMyc-p53^−/−^ tumor-bearing EPO-GEMM stomach. Representative image of n = 2 mice. (**n-p**) Representative hematoxylin & eosin (H&E) staining (N) and immunohistochemical staining for MYC (O) and E-cadherin (P) of a mMyc-p53^−/−^ tumor. Representative images of n = 2 mice. (**q**) Gross pathology image of a MYC-p53Q97* tumor-bearing EPO-GEMM stomach. Representative image of n = 1 mouse. (R) H&E staining of a MYC-p53Q97* tumor. Representative image of n = 1 mouse. (S) Sanger sequencing results confirming CRISPR base editing of the *Trp53* locus targeted by the indicated CRISPR/Cas9-sgRNA in a MYC-p53Q97* EPO-GEMM gastric tumor. Source data

The *MYC-p53*^*−/−*^ tumors could be detected by palpation and ultrasound imaging (upon reaching a size of ~3 mm) and they developed at the electroporation site through a stepwise progression of precursor lesions (Extended Data Fig. 2b–n) to moderately well-differentiated adenocarcinomas of the intestinal phenotype (Fig. 2b and Extended Data Fig. 3a–i)^9^. Initially, these tumors were predominantly (>50%) well differentiated (Extended Data Fig. 3a–c); at later stages, they transitioned from adenomatous to diffuse (Extended Data Fig. 3d–e) and eventually were almost entirely (>90%) composed of solid regions of poorly differentiated gastric carcinoma (Extended Data Fig. 3f–i). The well-differentiated areas expressed E-cadherin, CK8 and high levels of the proliferation marker Ki67, and they stained partially positive for mucin 6 and the parietal cell marker H^+^/K^+^ ATPase (Fig. 2b and Extended Data Fig. 3j–l). Of note, the same histological features were observed in tumors generated using the murine instead of the human *MYC* cDNA, confirming the use of human *MYC* as a valid approach, as established in GEMMs of other cancer types^19,24^ (Extended Data Fig. 3m–p). To further showcase the genetic flexibility of the platform, we used an optimized CRISPR base editor that can introduce precise single-nucleotide variants by C-to-T base substitution instead of indel-mediated gene knockout^25^. Introducing a recurrent but previously uncharacterized *p53* point mutation (Q97*) resulted in tumor generation, demonstrating the oncogenic function of this mutation and the compatibility of CRISPR base editing with the EPO-GEMM platform (Extended Data Fig. 3q–s).

**Extended Data Fig. 4:**
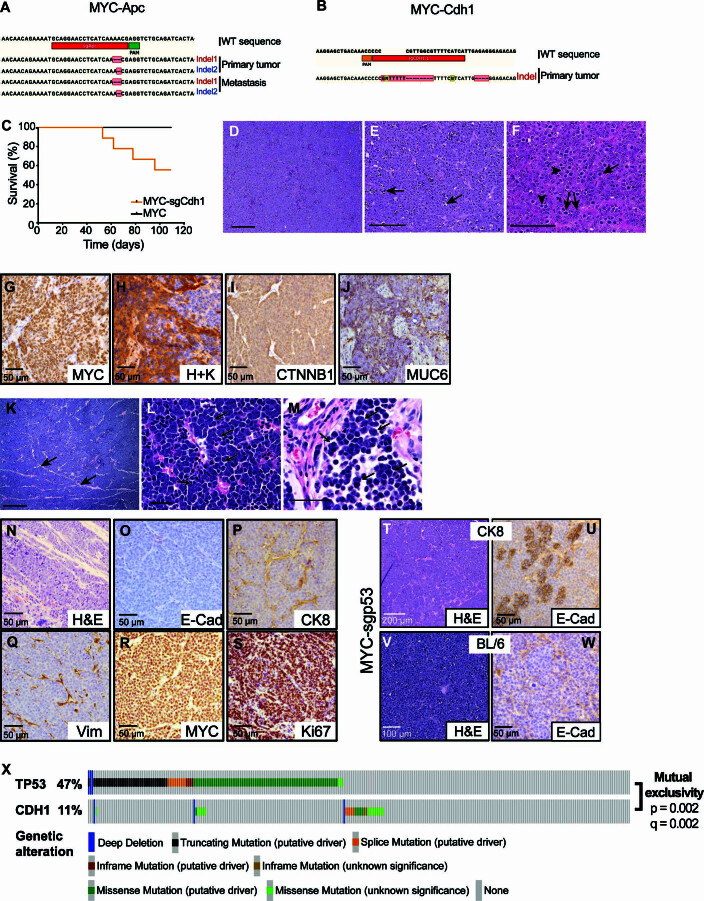
Histopathological analysis of GS gastric cancer EPO-GEMMs. (**a**) Sanger sequencing results confirming editing of the *Apc* locus targeted by the indicated CRISPR/Cas9-sgRNA in a MYC-Apc EPO-GEMM gastric tumor. (**b**) Sanger sequencing results confirming editing of the *Cdh1* locus targeted by the indicated CRISPR/Cas9-sgRNA in a MYC-Cdh1 EPO-GEMM gastric tumor. (**c**) Kaplan–Meier survival curves of C57BL/6 EPO-GEMMs after electroporation with either *MYC* and *Cdh1* vector (orange, n = 9 mice) or *MYC* vector only (black, n = 4 mice). (**d–f**) Histological analysis of a typical MYC-Apc EPO-GEMM gastric tumor: (D) MYC-Apc^−/−^ tumors were diffuse with sheets of neoplastic cells and little stroma (bar = 250 µm). Representative image of n = 3 mice. (E) Higher magnification of a MYC-Apc^−/−^ tumor with tightly cohesive neoplastic cells and scant extracellular matrix. There are abundant foci of apoptotic and necrotic cells (arrows) (bar = 250 µm). Representative image of n = 3 mice. (F) MYC-Apc^−/−^ tumors had abundant mitotic figures (arrows) and apoptotic cells (arrowheads) consistent with tumor growth and lack of differentiation (bar = 100 µm). Representative image of n = 3 mice. (**g–j**) Representative immunohistochemistry staining of a MYC-Apc EPO-GEMM gastric tumor for MYC (G), hydrogen/potassium ATPase (H+K) (H), beta-catenin (CTNNB1) (I), and MUC6 (J) Representative images of n = 10 mice. (**k-m**) Histological analysis of a typical MYC-Cdh1 EPO-GEMM gastric tumor: (K) MYC-Cdh1^−/−^ tumors showed diffuse histology with sheets of tumor cells subdivided by loose connective tissue (arrows) (bar = 250 µm). Representative image of n = 3 mice. (L) MYC-Cdh1^−/−^ tumor cells lacked normal cell-cell adhesion (arrows) consistent with loss of E-cadherin (bar = 100 µm). Representative image of n = 3 mice. (M) MYC-Cdh1^−/−^ liver metastases shared the poorly adherent individual cell feature (arrows) of the primary tumor (bar = 100 µm). Representative image of n = 3 mice. (**n-s**) H&E (N) and immunohistochemistry staining for E-Cadherin (E-cad) (O), cytokeratin 8 (CK8) (P), vimentin (Vim) (Q), MYC (R), and Ki67 (S) of a MYC-Cdh1 EPO-GEMM gastric tumor (bar = 50 µm). Representative images of n = 4 mice. (**t-u**) H&E (T) and immunohistochemistry for E-cadherin (E-Cad) (U) in a *MYC-p53*^−/−^ EPO-GEMM gastric tumor in a CK8-CreERT2; LSL-Cas9 mouse. Representative images of n = 6 mice. (**v, w**) H&E (V) and immunohistochemistry for E-cadherin (E-Cad) (W) of an undifferentiated *MYC-p53*^−/−^ EPO-GEMM gastric tumor in a C57BL/6 (BL/6) mouse. Representative images of n = 9 mice. (**x**) MSK-IMPACT oncoprint displaying the genomic status of alterations in *TP53* and *CDH1* in gastric cancer patients. Alterations in *p53* and *CDH1* are mutually exclusive in this setting. Statistical analysis via cBioPortal^71,72^. Source data

**Extended Data Fig. 5:**
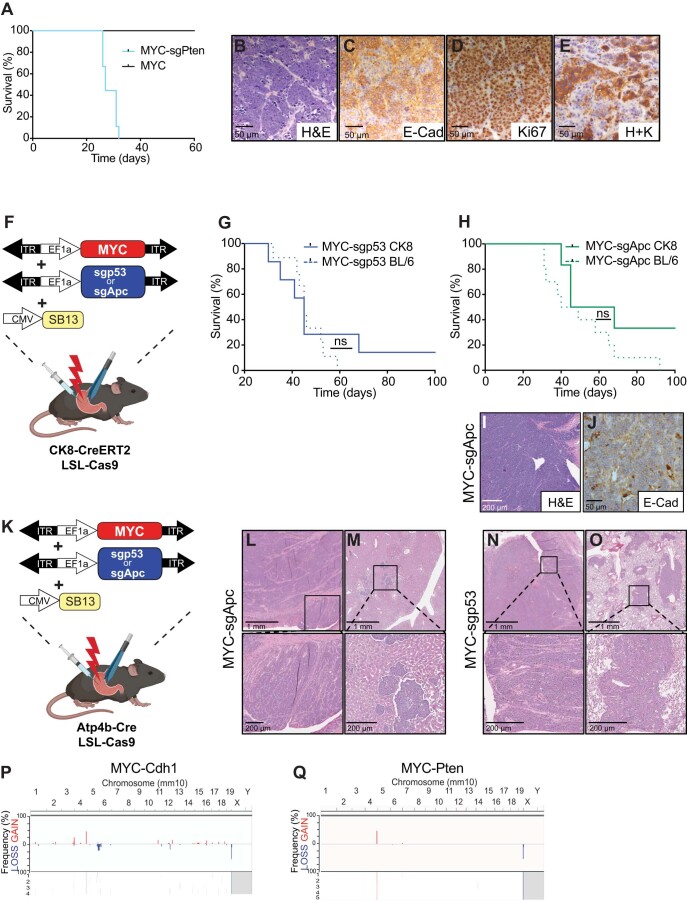
Gastric EPO-GEMM tumors originate from epithelial cells in the stomach. (**a**) Kaplan–Meier survival curves of C57BL/6 EPO-GEMMs after electroporation with either *MYC* and *Pten*^−/−^ vectors (blue, n = 9 mice) or *MYC* vector only (black, n = 4 mice). (**b–e**) Representative H&E (B) and immunohistochemistry staining for E-Cadherin (E-cad) (C), Ki67 (D), and hydrogen/potassium ATPase (H+K) (E) of a MYC-Pten EPO-GEMM gastric tumor. (**f**) Schematic of CK8-Cre restricted EPO-GEMM experiments. A transposon vector harboring *MYC* in combination with a Sleeping Beauty transposase (SB13) and/or a CRISPR-Cas9 vector targeting either *p53* or *Apc* were delivered into the stomach of CK8-CreERT2; LSL-Cas9 mice by direct *in vivo* electroporation. (**g**) Kaplan–Meier survival curves of C57BL/6 *MYC-p53*^−/−^ EPO-GEMMs (blue dashed line, n = 9 mice, same cohort as shown in Fig. 2a) or CK8-CreERT2; LSL-Cas9 *MYC-p53*^−/−^ EPO-GEMMs (blue line, n = 7 mice). (**h**) Kaplan–Meier survival curves of C57BL/6 *MYC-Apc*^−/−^ EPO-GEMMs (green dashed line, n = 10 mice, same cohort as shown in Fig. 2c) or CK8-CreERT2; LSL-Cas9 *MYC-Apc*^−/−^ EPO-GEMMs (green line, n = 6 mice). (**i-j**) H&E (I) and immunohistochemistry for E-cadherin (E-Cad) (J) of a CK8-CreERT2; LSL-Cas9 *MYC-Apc*^−/−^ EPO-GEMM gastric tumor. Representative images of n = 4 mice. (**k**) Schematic of Atp4b-Cre restricted EPO-GEMM experiments. A transposon vector harboring *MYC* in combination with a Sleeping Beauty transposase (SB13) and/or a CRISPR-Cas9 vector targeting either *p53* or *Apc* were delivered into the stomach of Atp4b-Cre; LSL-Cas9 mice by direct *in vivo* electroporation. (**l-m**) H&E of an Atp4b-Cre; LSL-Cas9 *MYC-Apc*^−/−^ EPO-GEMM gastric tumor (L) and a corresponding liver metastasis (M) (scale bar = 1 mm upper row, 200 µm lower row). Representative images of n = 2 mice. (**n-o**) H&E of an Atp4b-Cre; LSL-Cas9 *MYC-p53*^−/−^ EPO-GEMM gastric tumor (N) and a corresponding lung metastasis (O) (scale bar = 1 mm upper row, 200 µm lower row). Representative images of n = 2 mice. (**p**) Sparse whole-genome sequencing analysis of copy number alterations in MYC-Cdh1 gastric EPO-GEMM tumors (n = 4 mice). Frequency plot is shown on the top and individual sample tracks are provided on the bottom. (**q**) Sparse whole-genome sequencing analysis of copy number alterations in MYC-Pten gastric EPO-GEMM tumors (n = 5 mice). Frequency plot is shown on the top and individual sample tracks are provided on the bottom. Statistical analysis: (G, H) one-sided log-rank test. Source data

Next, we proceeded to model the GS subtype of gastric cancer. Because human GS tumors frequently harbor alterations in WNT pathway genes and/or *CDH1* (encoding E-cadherin) (Fig. 1b), we replaced the *p53* sgRNA with an sgRNA targeting *Apc* or *Cdh1* (Extended Data Fig. 4a,b). Delivery of these configurations to the gastric epithelium consistently produced tumors, with penetrance of 80% for MYC-sgApc and 40% for MYC-sgCdh1 and median survival of 44 and >110 d, respectively (Fig. 2c and Extended Data Fig. 4c). The *MYC-Apc*^−/−^ tumors had an undifferentiated histology. These tumors largely retained expression of the epithelial markers E-cadherin and CK8 and showed the expected stabilization of β-catenin and partial positivity for H^+^/K^+^ ATPase and mucin 6, as seen in human gastric cancer (Fig. 2d and Extended Data Fig. 4d–j). In contrast, the *MYC-Cdh1*^−/−^ tumors displayed undifferentiated histology along with the expected absence of E-cadherin expression and lack of normal cell–cell adhesion between tumor cells (Extended Data Fig. 4k–s). Notably, this diffuse undifferentiated histopathology resembled that in late-stage CIN tumors, which also became E-cadherin negative (Extended Data Fig. 4t–w). These observations suggest that p53-associated epithelial plasticity may be important in the evolution of CIN tumors. Accordingly, we found that *TP53* and *CDH1* mutations are mutually exclusive in samples from primary tumors of human patients with gastric cancer (Extended Data Fig. 4x). Finally, to illustrate how the range of tumor genotypes can be readily expanded, we generated EPO-GEMMs by knocking out the *Pten* tumor-suppressor gene, in accordance with the documented role of PI3K activating mutations in human gastric cancer^6–8^. The MYC-sgPten configuration produced tumors with high penetrance (80%) and median survival of 27 d post-electroporation; these tumors histologically resembled *MYC-Apc*^−/−^ tumors (Extended Data Fig. 5a–e).

Our histological observations are consistent with an epithelial cell of origin of the EPO-GEMM tumors, which was confirmed by generating tumors with comparable latency and presentation in a CK8-CreERT2; LSL-Cas9 host that restricts tumor initiation to the CK8^+^ epithelial compartment (Extended Data Fig. 4t–w and Extended Data Fig. 5f–j). Furthermore, given that CK8 is ubiquitously expressed in the epithelial compartment, we also used a Cre configuration that is restricted to the parietal cell lineage (Atp4b-Cre; LSL-Cas9), which is a potential cell of origin in gastric cancer^26–28^. Using both MYC-sgp53 and MYC-sgApc genotypes resulted in tumor formation with similar histological features to those of our non-Cre-restricted tumors, consistent with an epithelial and potentially parietal cell of origin in the EPO-GEMMs (Extended Data Fig. 5k–o).

Last, we characterized the degree of CIN of the tumors. Of note, *MYC-p53*^−/−^ but not *MYC-Apc*^−/−^, *MYC-Cdh1*^−/−^ or *MYC-Pten*^−/−^ tumors harbored recurrent genomic rearrangements, consistent with the CIN subtype of human gastric cancer (Fig. 2e–f and Extended Data Fig. 5p,q). Taken together, our data establish gastric cancer EPO-GEMMs as fast and flexible models that recreate fundamental histological and molecular features of the CIN and GS subtypes of the human disease.

### Model of MSI gastric cancer

The MSI subtype of gastric cancer, characterized by an increased frequency of mutations^11,29^ and a particular base substitution signature^30^, has not been recapitulated using traditional GEMMs^6,8^ (Fig. 1b). To generate such models, we combined the *MYC* transposon–transposase system with a CRISPR vector co-targeting *p53* and the mismatch repair gene *Msh2*. This approach allows for direct comparison of isogenic MSI (*MYC-p53*^−/−^*-Msh2*^−/−^) and microsatellite stable (MSS) (*MYC-p53*^−/−^) gastric cancers.

**Fig. 3:**
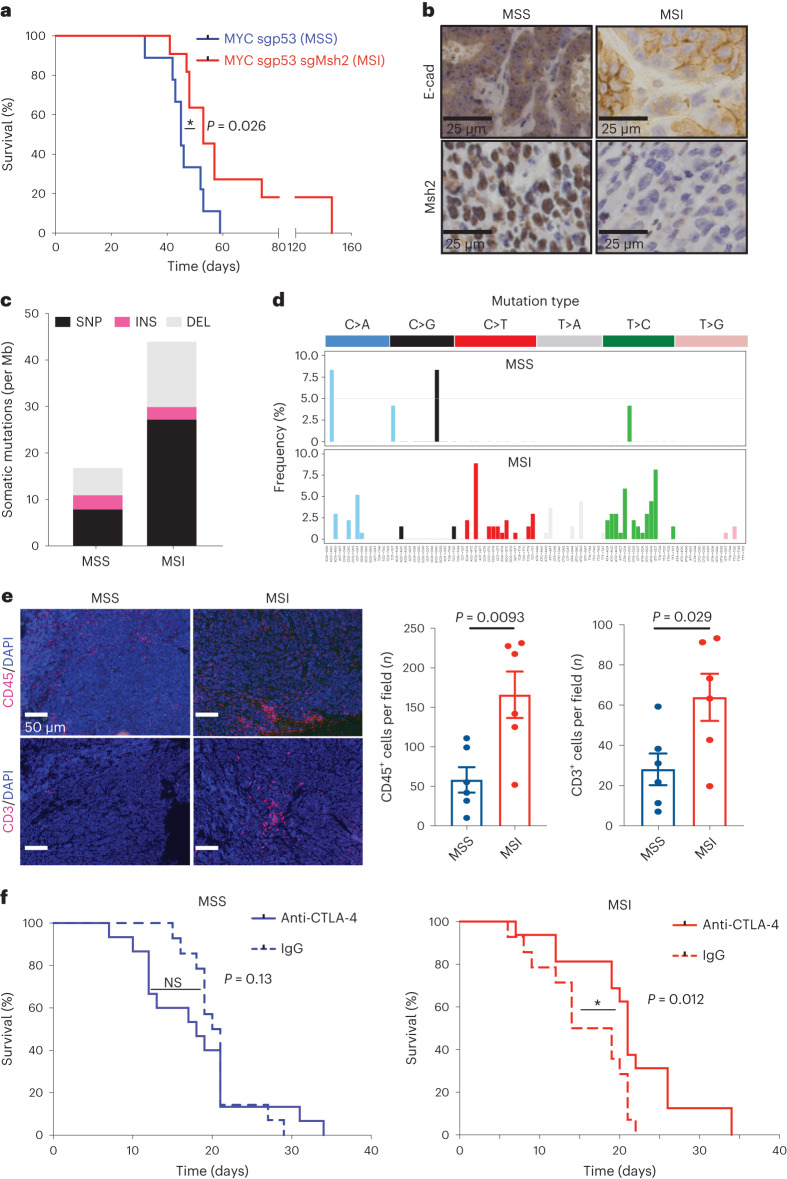
Somatic loss of *Msh2* induces microsatellite instability gastric cancer in mice. **a**, Kaplan–Meier survival curves of C57BL/6 EPO-GEMMs with either *MYC-p53*^−/−^ (MSS; same cohort as shown in Fig. 2a; blue, *n* = 9 mice) or *MYC-p53*^−/−^*-Msh2*^−/−^ (MSI; red, *n* = 11 mice) gastric cancer. **b**, Immunohistochemical staining for E-cadherin and Msh2 of *MYC-p53*^−/−^ (MSS) or *MYC-p53*^−/−^*-Msh2*^−/−^ (MSI) gastric EPO-GEMM tumors. Representative of *n* = 11 mice. **c**, WES analysis of somatic mutations per Mb in either *MYC-p53*^−/−^ or *MYC-p53*^−/−^*-Msh2*^−/−^ gastric EPO-GEMM tumors (*n* = 3 independent mice each). SNP, single-nucleotide polymorphism; INS, insertion; DEL, deletion. **d**, Base substitution signature in *MYC-p53*^−/−^ (MSS) and *MYC-p53*^−/−^*-Msh2*^−/−^ (MSI) gastric EPO-GEMM tumors (*n* = 3 independent mice each). **e**, Representative immunofluorescence staining of intratumoral regions of *MYC-p53*^−/−^ (MSS) or *MYC-p53*^−/−^*-Msh2*^−/−^ (MSI) gastric EPO-GEMM tumors for CD45 (red, top) or CD3 (red, bottom). Quantification to the right (*n* = 6 independent mice each). Data are presented as mean ± s.e.m. **f**, Kaplan–Meier survival curves of C57BL/6 gastric cancer EPO-GEMMs of either *MYC-p53*^−/−^ genotype (left) (*n* = 14 IgG-treated mice and *n* = 15 9H10-treated mice) or *MYC-p53*^−/−^*-Msh2*^−/−^ genotype (right) (*n* = 14 IgG-treated mice and *n* = 16 9H10-treated mice) after antibody-mediated blockade of CTLA-4 (9H10, 200 µg) (solid line) or IgG control (dashed line). Treatment was initiated (day 0) after tumor formation was confirmed by abdominal palpation. Statistical analyses were one-sided log-rank test (**a**,**f**) and unpaired *t*-test (**e**). NS, not significant; *P <0.05. Source data

**Extended Data Fig. 6:**
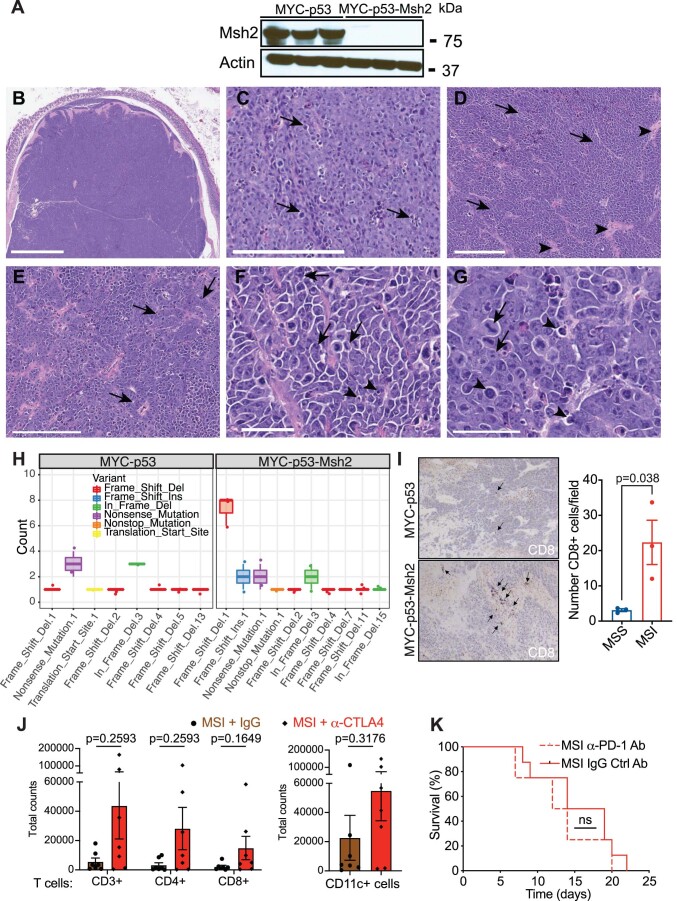
Histopathological and molecular analysis, and response to immunotherapy in MSI gastric cancer EPO-GEMMs. (**a**) Immunoblot of MSH2 and actin (loading control) in MSI or MSS gastric cancer cell lines (n = 3 lines each, derived from independent mice). (**b**) Most of the MSI tumor was typically diffuse (bar = 1 mm). Representative image of n = 3 mice. (**c**) Higher magnification of an MSI tumor with tightly cohesive neoplastic cells and scant extracellular matrix. There were abundant foci of apoptotic and necrotic cells (arrows) (bar = 250 µm). Representative image of n = 3 mice. (**d**) A region of diffuse phenotype in an MSI tumor composed of sheets of tumor cells (arrows) and minimal stroma (arrowheads) (bar = 200 µm). Representative image of n = 3 mice. (**e**) In rare areas, MSI tumors formed primitive glandular-like structures (arrows) (bar = 200 µm). (**f-g**) MSI tumors had numerous mitotic figures (arrows) and apoptotic cells (arrowhead) (bar = 50 µm). Representative images of n = 3 mice. (**h**) Whole-exome sequencing analysis of insertions (INS) or deletions (DEL) in either *MYC-p53*^−/−^ or *MYC-p53*^−/−^*-Msh2*^−/−^ gastric tumors (n = 3 independent mice each). The center horizontal line denotes the median (50^th^ percentile) value; box extends from the 25th to the 75th percentile of each group′s distribution of values. The whiskers mark the 5th and 95th percentiles. (**i**) Representative immunohistochemistry staining of intratumoral regions of *MYC-p53*^−/−^ (MSS) or *MYC-p53*^−/−^*-Msh2*^−/−^ (MSI) gastric EPO-GEMM tumors for CD8. Quantification to the right (n = 3 independent mice each). Data are presented as mean values +/− s.e.m. (**j**) Number of CD3+, CD4+ and CD8+ T cells (left) or CD11c+ cells (right) in MSI gastric tumors after treatment of mice with antibodies targeting CTLA-4 or IgG control (n = 7 independent mice each). Statistical analysis by two-tailed Mann–Whitney *U-*test. Data are presented as mean values +/− s.e.m. (**k**) Kaplan–Meier survival curves of C57BL/6 gastric cancer EPO-GEMMs of *MYC-p53*^−/−^*-Msh2*^−/−^ genotype (n = 8 mice IgG-treated, 4 mice α-PD-1-treated) after antibody-mediated blockade of PD-1 (RMP1-14, 200 µg) (solid line) or IgG control (dashed line). Treatment was initiated (day 0) after tumor formation was confirmed by abdominal palpation. Statistical analysis by one-sided log-rank test; ns = not significant (*P* > 0.05). Source data

Consistent with the less-aggressive nature of MSI compared to MSS tumors in human patients with gastric cancer^6^, the median survival of mice electroporated with *Msh2* sgRNAs was longer than that of *MYC-p53*^−/−^ controls (53 versus 45 d, respectively; 73% penetrance; Fig. 3a). Of note, the tumors resulting from MYC-sgp53-sgMsh2 EPO harbored genetic alterations of the *Msh2* locus and lacked MSH2 expression in the tumor (Fig. 3b and Extended Data Fig. 6a), so despite the extended survival, *Msh2* disruption seemed to confer a selective advantage during tumorigenesis. These MSI tumors again displayed a mixture of well-differentiated E-cadherin-expressing adenocarcinoma and poorly differentiated gastric carcinoma at late-stage disease (Fig. 3b and Extended Data Fig. 6b–g). Furthermore, whole-exome sequencing (WES) revealed a significantly higher number of genetic alterations in MSI versus MSS tumors, mainly consisting of single-nucleotide variants, small indels (mostly of a single base pair) and a C > T- and T > C-dominated base substitution signature consistent with human MSI cancers^30^ (Fig. 3c–d and Extended Data Fig. 6h).

The high mutational burden of MSI tumors typically results in abundant tumor neoantigens presented on major histocompatibility complex class I molecules that can facilitate a T cell-mediated antitumor response^11^ and contribute to increased tumor responsiveness to immunomodulatory drugs^10,12,13,31^. Accordingly, MSI EPO-GEMMs had more infiltrating CD45^+^ and CD3^+^ cells (consisting mostly of CD8^+^ T cells) than their MSS counterparts, albeit with substantial intratumoral heterogeneity, possibly reflecting the random process of generating immunogenic neoantigens^32^ (Fig. 3e and Extended Data Fig. 6i). Furthermore, neither MSS nor MSI EPO-GEMMs responded to anti-PD-1 checkpoint blockade, mimicking the results observed in other MSI mouse models^32^, yet MSI tumors responded to anti-CTLA-4 checkpoint blockade (Fig. 3f and Extended Data Fig. 6j,k). This observation could reflect the different mechanisms of action of these immunomodulating agents^33^. Taken together, these results show that MSI EPO-GEMMs largely recapitulate the genetic and immune-microenvironmental patterns of human MSI gastric cancers.

### Transcriptomic features of gastric cancer EPO-GEMMs

**Fig. 4:**
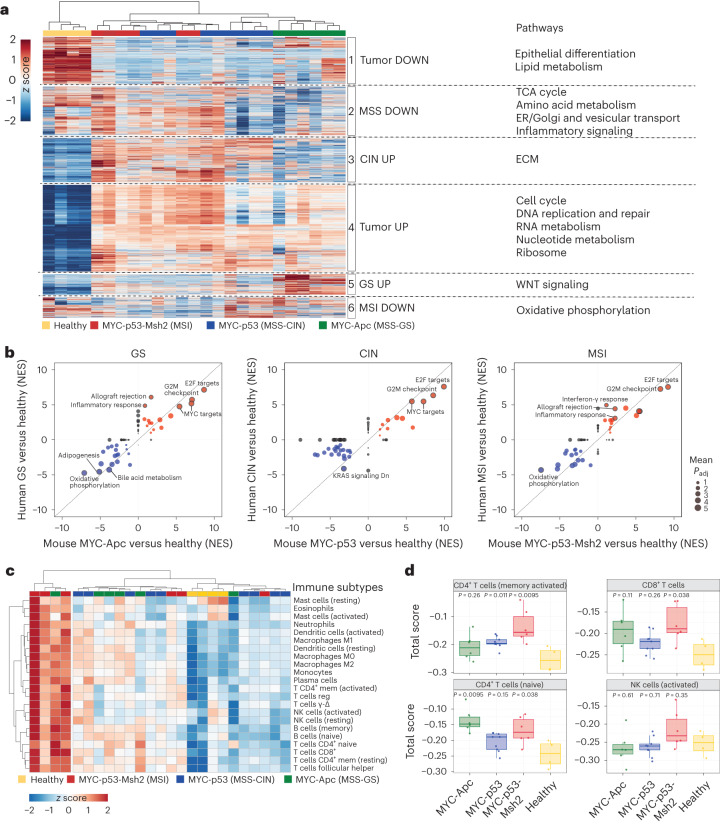
EPO-GEMMs recapitulate transcriptional features of human gastric cancer subtypes. **a**, Heat map of DEGs across the indicated EPO-GEMM samples (each column represents one mouse; healthy *n* = 4, MSI *n* = 6, MSS-CIN *n* = 9, MSS-GS *n* = 6 independent samples derived from separate mice). Hierarchical clustering segregated all samples based on six signatures (1–6). Key pathways enriched in each signature are shown on the right. Complete lists of genes and pathway predictions are provided in Supplementary Table 1. TCA, tricarboxylic acid; ER, endoplasmic reticulum. **b**, Comparison of GSEA NES for Hallmark pathways enriched in EPO-GEMM (*x* axis) and human (*y* axis) tumors versus healthy stomach for the indicated genotypes/subtypes. Key pathways are highlighted. Circle size represents the adjusted *P* value. Complete lists of pathways and NES scores are provided in Supplementary Tables 2–4. **c**, Heat map of CIBERSORT signatures for distinct immune subpopulations in the indicated EPO-GEMM tumor and healthy gastric samples. **d**, Boxplots of CIBERSORT signature scores for the indicated immune populations and EPO-GEMM tumors ((*n* = 9 (*MYC-p53*^−/−^), *n* = 6 (*MYC-Apc*^−/−^), *n* = 6 (*MYC-p53*^−/−^*-Msh2*^−/−^) and *n* = 4 (healthy stomach) independent samples derived from separate mice). The center horizontal line denotes the median (50th percentile) value; the box extends from the 25th to the 75th percentile of each group’s distribution of values. The whiskers mark the 5th and 95th percentiles. Complete lists of CIBERSORT signature scores are provided in Supplementary Table 5. Two-sided Wilcoxon signed-rank test. Source data

Human gastric tumors exhibit gene expression patterns that reflect features of their molecular classification^8^. Hence, we performed bulk RNA sequencing on tumors from EPO-GEMMs that represent the GS, CIN and MSI subtypes, as well as on healthy gastric tissue. Hierarchical clustering of all samples indicated that tumor genotype was the most prominent factor dictating the transcriptional landscape of different tumors (Fig. 4a). Consistent with human data^8^ and the role of *p53* loss in increasing plasticity^34^ (Fig. 2b and Extended Data Fig. 4), CIN tumors showed the greatest inter-tumoral heterogeneity. We performed Gene Ontology analysis of six clusters that segregated differentially expressed genes (DEGs) across all samples; this analysis revealed transcriptional features that were either tumor-universal or tended to group with specific tumor subtypes (Fig. 4a and Supplementary Table 1). First, as expected, proliferation-related pathways were enriched and differentiation-related pathways were depleted across all tumor samples. Second, MYC-Apc GS tumors showed a prominent WNT signaling signature, consistent with their *Apc*-null status. Third, CIN tumors exhibited a weak but statistically significant enrichment of extracellular matrix (ECM) genes, which may reflect *p53*-related ECM remodeling seen in other cancers^35–37^. Fourth, in agreement with our immune-focused analysis above, MSS tumors under-expressed genes involved in inflammatory signaling pathways, as well as genes involved in metabolism and vesicular transport. On the other hand, MSI tumors showed reduced expression of genes involved in oxidative phosphorylation, perhaps due to mitochondrial damage linked to mismatch repair deficiency^38,39^.

**Extended Data Fig. 7:**
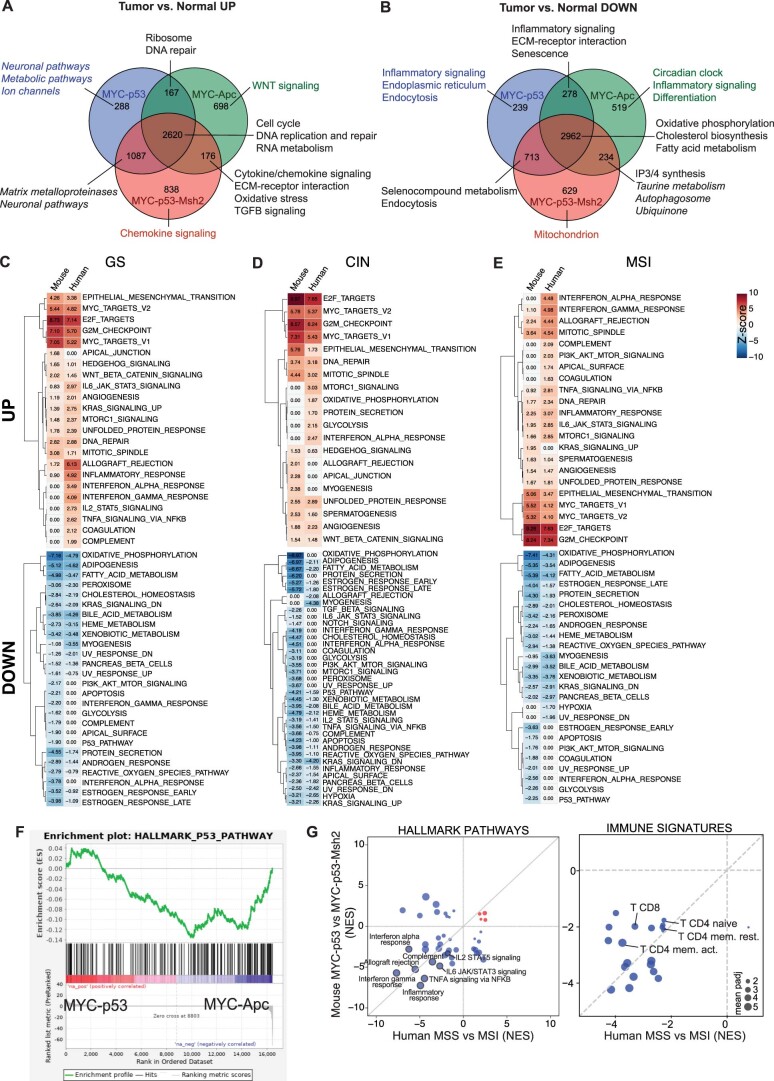
Transcriptomic analysis of gastric cancer EPO-GEMMs. (**a, b**) Venn diagrams showing overlap of differentially upregulated (A) or down regulated (B) genes (vs. healthy stomach) for the indicated EPO-GEMM tumor genotypes. Key pathways enriched in each gene subset are labeled accordingly. Complete lists of pathway predictions are provided in Supplementary Tables 6–19. (**c–e**) Complete lists of the Hallmark Pathways and NES scores shown in Fig. 4b, along with the NES scores for human gastric tumors of the corresponding subtypes. (**f**) Gene set enrichment analysis (GSEA) for Hallmark p53 Pathway comparing *MYC-p53*^−/−^ and *MYC-Apc*^−/−^ gastric tumors. (**g**) Comparison of GSEA NES scores for hallmark pathways (left) or immune populations (right) enriched in mouse (*y* axis) and human (*x* axis) MSI gastric tumors. Highlighted are key immune populations enriched in MSI tumors. Circle size represents -log(adjusted *P* value). A complete list of NES scores is provided in Supplementary Tables 20, 21. Source data

These observations were reinforced by Gene Ontology analysis of shared and unique DEGs for the distinct tumor genotypes, which highlighted a relative depletion of p53 signatures in *MYC-p53*^−/−^ tumors and enrichment of immune-related pathways in MSI tumors (Extended Data Fig. 7a–f and Supplementary Tables 6–19). In addition, genes related to the epithelial-to-mesenchymal transition were upregulated to various degrees in all tumor subtypes, suggesting high invasive and metastatic potential (Extended Data Fig. 7c–e). Notably, the transcriptional features of EPO-GEMM tumors correlated with those of human gastric tumors of the respective subtypes, which was largely driven by dominant MYC, proliferation and immune-related signatures (Fig. 4b, Extended Data Fig. 7c–e and Supplementary Tables 2–4).

To assess immune cell infiltrates in different tumor subtypes, we employed the CIBERSORT^40^ algorithm to identify immune cell signatures in bulk tumor samples. Hierarchical clustering segregated a subset of MSI tumors (3 of 6 samples) as broadly enriched in most immune signatures (Fig. 4c), including CD4^+^ and CD8^+^ T cells (Fig. 4d and Supplementary Table 5). Of note, the immune cell infiltrates and associated signaling pathways displayed similarities between murine and human MSS versus MSI tumors, with the MSI tumors showing increased expression of inflammatory pathways and most immune cell signatures (Extended Data Fig. 7g and Supplementary Tables 20 and 21). Overall, these gene expression data further demonstrate the molecular fidelity of EPO-GEMMs to their human counterparts and identify pathways that may underlie both common and unique features of gastric cancer subtypes.

### Metastatic organotropism in gastric cancer EPO-GEMMs

**Fig. 5:**
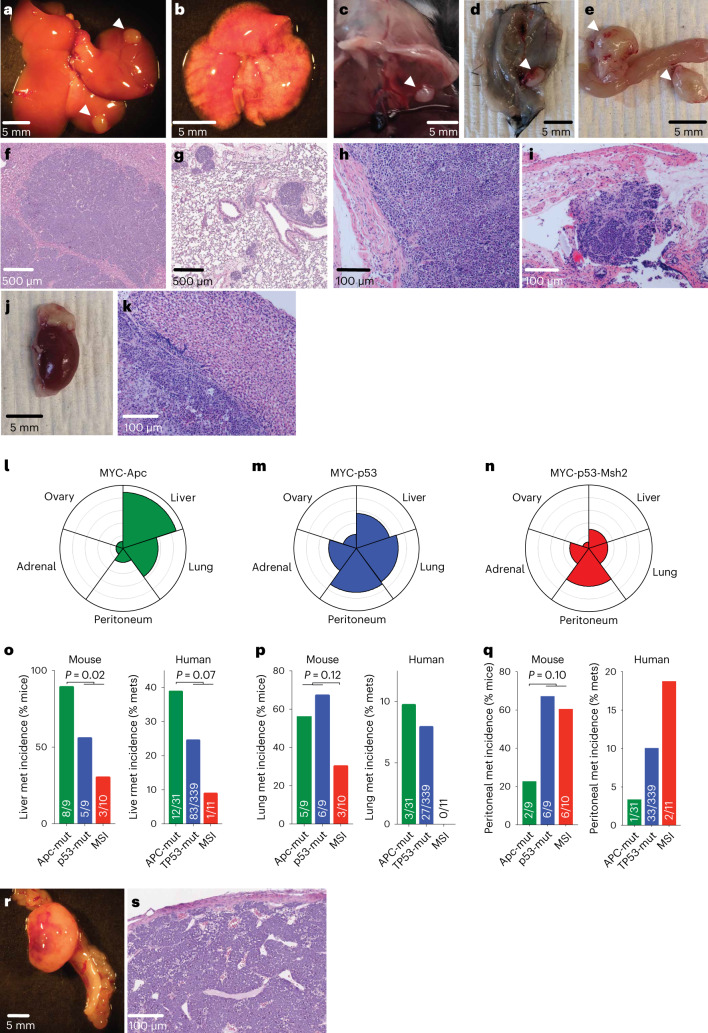
Gastric cancer EPO-GEMMs display metastatic patterns that recapitulate the human disease. **a**–**e**, Representative gross pathology images of liver (**a**), lungs (**b**) and peritoneal metastases at the diaphragm (**c**), body wall (**d**) and abdomen (**e**) from *MYC-p53*^−/−^ gastric cancer EPO-GEMMs. Arrows point to macroscopic tumors. **f**–**i**, Representative H&E-stained histological images of liver (**f**), lung (**g**) and peritoneal (**h**,**i**) metastases from *MYC-p53*^−/−^ gastric cancer EPO-GEMMs. **j**,**k**, Representative macroscopic (**j**) and H&E-stained histological (**k**) images of an adrenal metastasis from *MYC-p53*^−/−^*-Msh2*^−/−^ gastric cancer EPO-GEMMs. **l**–**n**, Petal plots of metastasis incidence in the specified organs of EPO-GEMMs harboring the indicated genotypes. The radius of each petal corresponds to the fraction of mice developing metastases (mets) in the indicated organ; the outermost ring corresponds to 100% (*n* = 9–10 independent mice). Detailed numbers are provided in the statistical source data. **o–q**, Incidence of liver (**o**), lung (**p**) or peritoneal metastasis (**q**) in EPO-GEMMs and in the MSK-IMPACT cohort of patients with esophagogastric cancer with the corresponding genetic alterations. The exact number of independently analyzed tumors is indicated. Statistical analysis by Fisher’s exact test. **r–s**, Representative macroscopic (**r**) and H&E-stained histological (**s**) images of an ovarian metastasis from a *MYC-p53*^−/−^ gastric cancer EPO-GEMM. Source data

**Extended Data Fig. 8:**
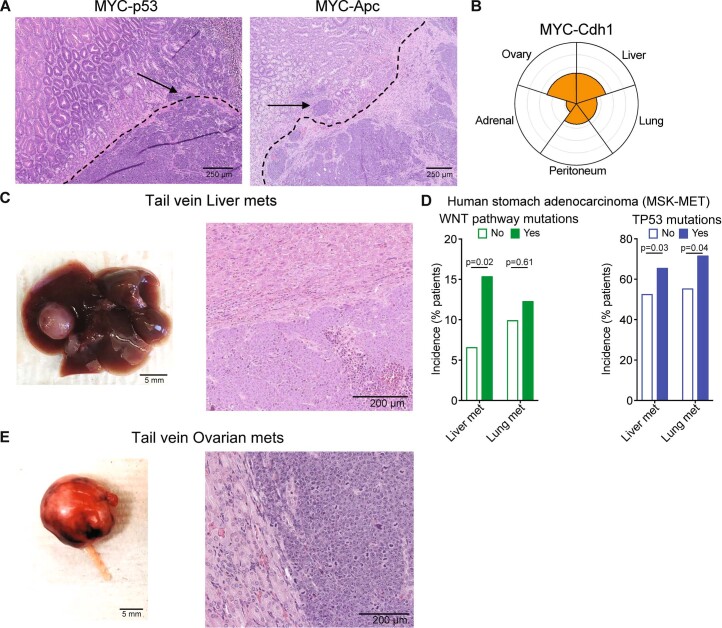
Invasive and metastatic features of gastric cancer EPO-GEMMs. (**a**) H&E images showing the boundary (dashed lines) between normal stomach and gastric tumors with areas of local invasion (arrows) in C57BL/6 EPO-GEMMs with *MYC-p53*^−/−^ (left) and *MYC-Apc*^−/−^ (right). Representative image of n = 3 mice. (**b**) Petal plot of metastasis incidence in the specified organs of MYC-Cdh1 EPO-GEMMs. The radius of each petal corresponds to the fraction of mice developing metastases in the indicated organ; the outermost ring corresponds to 100% (n = 6 independent mice). (**c**) Macroscopic (left) and H&E histology image (right) of liver metastasis in mice subjected to tail vein injection of a *MYC-Apc*^−/−^ gastric cancer cell line. Images are representative of 3 metastasis-bearing livers from 10 mice analyzed. (**d**) Incidence of liver and lung metastases among the MSK-MET cohort of gastric cancer patients with WNT pathway mutations or *TP53* mutations. Statistical analysis as reported^42^. (**e**) Macroscopic (left) and H&E histology image (right) of ovarian metastasis in mice subjected to tail vein injection of *MYC-p53*^−/−^ gastric cancer cell lines. Images are representative of one metastasis-bearing ovary from 10 mice analyzed for each of three independent cell lines. Source data

Perhaps the most clinically important feature of gastric cancer is its propensity to metastasize, a property that is rarely observed in traditional gastric cancer GEMMs^16,18^. In contrast, gastric cancer EPO-GEMMs were invasive and reproducibly metastasized to the liver, lungs, peritoneum and adrenal glands (Fig. 5a–k and Extended Data Fig. 8a,b), as frequently observed in patients; however, the organotropism of metastases differed across genotypes. Mice harboring *Apc*-null GS tumors showed a higher frequency of liver metastasis (8 of 9, 88% of mice) compared to those harboring *p53*-null CIN tumors (5 of 9, 56% of mice) or *Msh2*-null MSI tumors (3 of 10, 30% of mice) (Fig. 5l–o). Notably, the capacity of *Apc*-null GS tumors to colonize the liver was also noted following introduction of an *Apc*-null tumor-derived line via tail vein injection, an experimental metastasis assay that strongly favors seeding to the lung (Extended Data Fig. 8c). In contrast, *p53*-null tumors (whether MSS or MSI) showed markedly more peritoneal metastasis (6 of 9, 67% of mice for MSS, and 6 of 10, 60% of mice for MSI, compared to 2 of 9, 22% of mice for GS tumors), whereas MSI tumors showed markedly less lung metastasis (3 of 10, 30% of mice, compared to 5 of 9, 56% of mice for GS and 6 of 9, 67% mice for CIN tumors) (Fig. 5l–n,p–q).

The different metastatic profiles of gastric cancer subtypes in the EPO-GEMMs were unexpected. To determine whether similar patterns exist in the human disease, we analyzed clinically annotated MSK-IMPACT^41^ data (Fig. 5o–q). Indeed, liver metastases accounted for 39% (12 of 31) of metastases that harbored *APC* mutations, compared to 24% (83 of 339) and 9% (1 of 11) of metastases harboring *TP53* or mismatch repair mutations, respectively (Fig. 5o). Likewise, none (0 of 11) of the mismatch repair-mutant metastases was derived from the lung, in contrast to 10% (3 of 31) and 8% (27 of 339) of *APC*- and *TP53*-mutant metastases, respectively (Fig. 5p). Finally, only 3% (1 of 31) of *APC*-mutant metastases were of peritoneal origin, compared to 10% (33 of 339) and 18% (2 of 11) of *TP53*-mutant and MSI metastases, respectively (Fig. 5q). Corroborating these results, WNT pathway alterations were significantly associated with liver but not lung metastasis, whereas mutations in *TP53* were associated with small increases in the metastasis incidence to both organs (Extended Data Fig. 8d)^42^.

Some patients with gastric cancer develop ‘Krukenberg tumors’^43,44^, which arise from metastasis to the ovary. These poorly understood tumors often occur in young women (median age 45 years) and confer a dismal prognosis^45^. Owing in part to the lack of model systems, the etiology of these tumors remains unresolved and there is an ongoing debate as to whether the cancer cells spread from the primary tumor through a peritoneal, lymphatic or hematogenous route^45^. Remarkably, 10–20% of gastric cancer EPO-GEMMs developed metastases to the ovaries, irrespective of genotype (Fig. 5r,s). The capacity for ovarian metastasis was maintained in primary tumor lines (1 of 3 lines per genotype) assayed by tail vein injection (Extended Data Fig. 8e). These data indicate that, while other routes are possible, dissemination through the bloodstream is a viable route for gastric cancer metastasis to the ovaries. Together, these data highlight the relevance of EPO-GEMMs as a robust platform to study metastatic gastric cancer and suggest a role for tumor genotype in metastatic organotropism.

### Natural killer cells suppress gastric cancer metastasis

**Fig. 6:**
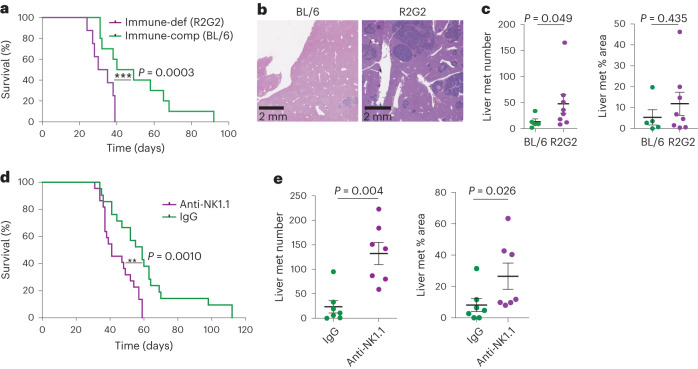
NK cells suppress gastric cancer metastasis. **a**, Kaplan–Meier survival curves of immunocompetent C57BL/6 mice (BL/6, green; same cohort as shown in Fig. 2c) and immunodeficient R2G2 mice (purple, *n* = 8 mice) with electroporation-induced *MYC-Apc*^−/−^ gastric cancer. **b**, H&E staining of liver metastases from mice in **a**. Representative of *n* = 5 mice (BL/6) or *n* = 8 mice (R2G2) per group. **c**, Quantification of the number of liver metastases (left) and the percentage area of total liver occupied by metastases (right) from a subset of mice in **a** (BL/6 *n* = 5 mice; R2G2 *n* = 8 mice). **d**, Kaplan–Meier survival curves of BL/6 *MYC-Apc*^−/−^ gastric cancer EPO-GEMMs treated with an NK1.1-targeting antibody (purple, *n* = 22 mice) or IgG control (green, *n* = 21 mice). **e**, Quantification of the number of liver metastases (left) and the percentage area of total liver occupied by the metastases (right) in a randomly chosen subset of mice from **d** (IgG *n* = 7 mice; NK1.1 *n* = 7 mice). Statistical analyses were one-sided log-rank test (**a**,**d**) and two-tailed Mann–Whitney *U*-test (**c**,**e**). Source data

Studies using carcinogen-induced or transplantation models have revealed roles for immune cells in both facilitating and limiting metastatic spread, but little is known about the influence of the immune system on metastasis in autochthonous, genetically defined models that most closely resemble the human scenario^46,47^. Thus, we harnessed the host flexibility of the EPO-GEMM approach to engineer *MYC-Apc*^−/−^ tumors in two recipient strains: (1) wild-type C57BL/6 mice, which are fully immune-competent; or (2) Rag2-Il2rg double-knockout (R2G2) mice, which are deficient in T, B and natural killer (NK) cells and have reduced levels of neutrophils, macrophages and dendritic cells. Tumor-bearing R2G2 mice showed reduced survival and a greater incidence of liver metastasis compared to immunocompetent recipients (Fig. 6a–c). These data indicate that the immune system suppresses metastasis as tumors develop in an autochthonous gastric cancer model.

**Extended Data Fig. 9:**
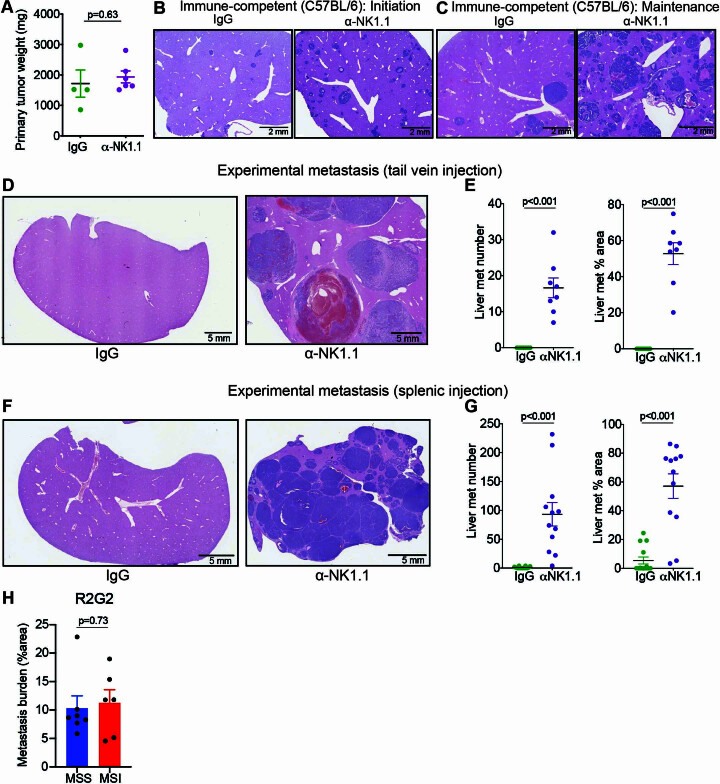
NK cells suppress gastric cancer metastasis to the liver. (a) Primary tumor weights of a random subset of mice from Fig. 6d (IgG n = 4 mice; α-NK1.1 n = 6 mice). (**b**, **c**) Hematoxylin and eosin (H&E) staining of liver metastases of C57BL/6 *MYC-Apc*^−/−^ EPO-GEMMs treated with an NK1.1-targeting antibody or the respective IgG control directly before tumor initiation (B) (representative image of n = 7 mice) or after palpable tumor formation (C) (representative image of n = 8 mice). (**d**) H&E staining of livers of C57BL/6 mice after tail vein injection of *MYC-Apc*^−/−^ gastric cancer cells and treatment with either an antibody targeting NK1.1 (right) or IgG control (left) (representative images of n = 8–9 mice per group). (**e**) Quantification of the number of liver metastases (left) and the percentage area of total liver occupied by the metastasis (right) from mice in (D) (n = 8 independent mice). (**f**) H&E staining of livers of C57BL/6 mice after splenic injection of *MYC-Apc*^−/−^ gastric cancer cells and treatment with either an antibody targeting NK1.1 (right) or IgG control (left). Representative images of n = 12–14 mice per group. (**g**) Quantification of the number of liver metastases (left) and the percentage area of total liver occupied by the metastases (right) from mice in (F) (n = 12 independent mice). (**h**) Quantification of the percentage area of total lung occupied by metastases in a randomly chosen subset of R2G2 mice from Fig. 7b (MSS n = 7 mice; MSI n = 6 mice). Statistical analysis: (A), (E), (G), (H) two-tailed Mann–Whitney *U-*test. Data are presented as mean values +/− s.e.m. Source data

NK cells are a prominent immune cell type that can limit metastasis in certain transplantation-based models^48^. To assess the role of NK cells in an autochthonous context, we administered NK1.1-targeting antibodies in immunocompetent mice from the time of electroporation, which results in systemic depletion of NK and related cells^47^. These mice displayed decreased overall survival and increased liver metastasis compared to isotype-treated controls (Fig. 6d,e); however, primary tumor size at end point did not differ (Extended Data Fig. 9a), suggesting that the contribution of NK cells to survival was mainly due to the suppression of metastasis. Reinforcing this point, results were similar when NK cells were depleted only after detection of a palpable primary tumor (Extended Data Fig. 9b,c) or when we examined metastatic potential of circulating tumor cells following tail vein or intrasplenic injection (Extended Data Fig. 9d–g). Notably, liver metastasis was more strongly enhanced by NK1.1-mediated depletion than by using R2G2 hosts, suggesting that non-NK immune cells that are absent in R2G2 mice (for example, neutrophils^46^) might promote metastatic spread in this setting. Taken together, these data show that NK cells play an important role in curtailing gastric cancer metastasis to the liver.

### CD8^+^ T cell surveillance of MSI gastric cancer metastasis

**Fig. 7:**
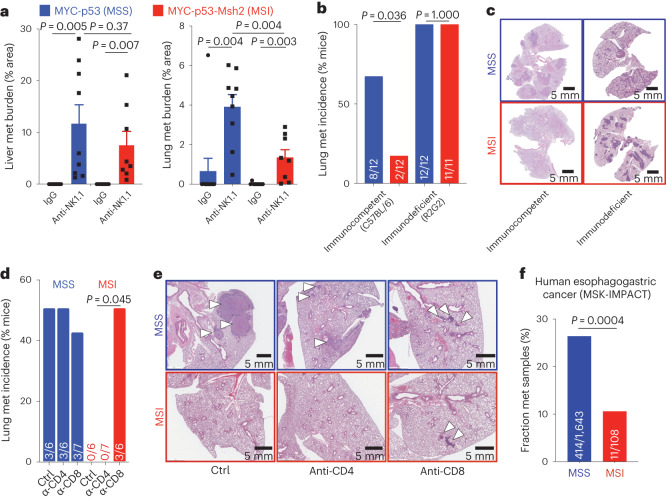
CD8^+^ T cells provide an added layer of metastasis immune surveillance in MSI tumors. **a**, Metastatic burden (% tumor area) in the liver (left) or lung (right) of BL/6 mice after tail vein injection of *MYC-p53*^−/−^ (MSS, blue, *n* = 9 or 10 mice) or *MYC-p53*^−/−^*-Msh2*^−/−^ (MSI, red, *n* = 8 or 9 mice) gastric cancer cells. Mice were treated with either an NK1.1-targeting antibody or IgG control. Data are presented as mean ± s.e.m. **b**, Incidence of lung metastasis after tail vein injection of MSS or MSI gastric cancer cells into immunocompetent (C57BL/6) or immunodeficient (R2G2) mice. Exact numbers of independent mice are indicated on each bar. **c**, H&E staining of lungs isolated from mice in **b**. Representative of *n* = 11 mice (MSI in R2G2) or *n* = 12 mice (all other conditions) per group. **d**, Incidence of lung metastasis after tail vein injection of MSS or MSI gastric cancer cells into immunocompetent (C57BL/6) mice that were treated with either CD4- or CD8-targeting antibodies or an IgG control. Exact numbers of independent mice are indicated on each bar. **e**, H&E images of lungs isolated from mice in **d**. Exact numbers of independent mice are indicated on each bar. **f**, Fraction of metastatic samples in the MSK-IMPACT cohort of patients with esophagogastric cancer with either MSS or MSI disease. The exact number of independently analyzed tumors is indicated on each bar. Statistical analyses were ordinary one-way ANOVA (**a**) and Fisher’s exact test (**b**,**d**,**f**) . Source data

To characterize how the immune system restricts metastatic spread across gastric cancer subtypes, we performed experimental metastasis assays using cell lines derived from primary EPO-GEMM tumors representing the GS (*MYC-Apc*^−/−^), CIN (*MYC-p53*^−/−^) and MSI (*MYC-p53*^−/−^*-Msh2*^−/−^) subtypes. Mice were treated with NK1.1 versus IgG control antibody starting 2 days before tail vein injection and metastasis development was assessed 3–4 weeks later. NK cell depletion led to a significant increase in both liver and lung metastatic burden in mice injected with either CIN (MSS) or MSI cancer cells (Fig. 7a); however, the metastatic potential of MSI tumors remained lower than that of MSS tumors even following NK cell depletion, an effect that reached statistical significance in the lung (Fig. 7a). Corroborating these results, only 17% (2 of 12) of immunocompetent mice injected with MSI gastric cancer cells developed overt lung metastases, compared to 75% (8 of 12) of mice injected with MSS tumor cells (Fig. 7b,c). Nevertheless, the MSI and MSS subtypes showed similar abilities to form lung metastases with comparable overall lung tumor burden following tail vein injection into immunodeficient R2G2 mice (Fig. 7b,c and Extended Data Fig. 9h), indicating that there was no appreciable difference in their cell-intrinsic potential to colonize the lung.

Because mismatch repair-deficient tumors can present high amounts of neoantigens and elicit T cell-mediated immune responses (Fig. 3c–f)^11^, we reasoned that cytotoxic T cells may also contribute to the surveillance of MSI tumors. We therefore depleted either CD4^+^ or CD8^+^ cells and assessed the metastatic potential of either MSS or MSI tumor cells following tail vein injection. Whereas MSS cells formed metastases across all conditions, MSI cells seeded metastases only in the CD8-depleted condition (Fig. 7d,e). These results are consistent with reduced metastasis incidence that we estimated in MSI patients of the MSK-IMPACT cohort (Fig. 7f). In sum, these data reveal a bimodal surveillance of gastric cancer metastasis, a genotype-agnostic control by NK cells supplemented with MSI-specific control by CD8^+^ T cells.

## Discussion

Here we present a suite of fully somatic mouse models of gastric cancer, termed gastric cancer EPO-GEMMs, produced by delivery of genetic elements directly to the stomach using tissue electroporation. By combining different mutational events associated with distinct tumor subtypes, we demonstrate that this platform can produce models of all three nonviral molecular subtypes of human gastric cancer. These models mirror defining histological and transcriptional properties of their respective human subtypes and present similar patterns of chromosomal (in)stability and mutational signatures. Moreover, each model reproducibly metastasizes to clinically relevant anatomical sites. These features demonstrate the relevance of gastric cancer EPO-GEMMs for discovery and preclinical studies, including in the context of metastasis.

The genetic flexibility of the gastric cancer EPO-GEMMs eliminates the need for extensive strain intercrossing and enables rapid testing of any genetic combination by simply changing the sequence of electroporated constructs. Moreover, synchronous cohorts of animals that will develop genotypically defined tumors can be produced in a day, thereby greatly simplifying the execution of mechanistic and preclinical studies. Thus, gastric cancer EPO-GEMMs offer advantages over carcinogen-induced models, which do not produce genetically defined tumors^49,50^ and Cre/lox-based models, which are limited to available germline strains, yield asynchronous cohorts and entail substantial animal waste as unavoidable byproducts of strain intercrossing^16,17^. Furthermore, EPO-GEMMs generate focal cancers in adult mice, avoiding the confounding effects of tissue-wide gene activation/inactivation during embryogenesis or, conversely, the requirement for tamoxifen (which can induce gastric metaplasia^51,52^) to recombine germline alleles in adulthood. Finally, EPO-GEMMs offer the unique capability to readily change the host, which provides a flexible and robust platform to study tumor–host interactions. Given these features, EPO-GEMMs are well poised for use in the evaluation of new therapeutic strategies based on genetic and molecular biomarkers (extending beyond MSI status), a potentially valuable capability as such biomarkers are becoming prevalent in the evolving landscape of gastric adenocarcinoma research and treatment^53,54^.

A notable limitation of the EPO-GEMM approach is that it does not provide precise control over the cell of origin for tumor development and could induce somatic alterations in stromal cells. Nevertheless, we verified that tumors originate from the epithelial compartment and provided evidence that they originate specifically from parietal cells, supporting previous findings that gastric cancer can originate from these cells^26–28^. Furthermore, previous work using direct in vivo electroporation of CRISPR-Cas9 vectors to model pancreatic cancer did not detect somatic alterations in normal cells by ultra-deep sequencing, suggesting that these events are very rare at best and thus unlikely to influence the course of tumorigenesis^22^. In addition, the engineering of multiple alleles using pooled plasmids entails the risk of introducing intratumoral heterogeneity, which should be taken into consideration and could potentially be monitored by using stable constructs linked to fluorescent reporters. Finally, the injection technique may in rare cases lead to a breaching of the mucosal muscle layer, thereby artificially facilitating metastatic spread.

With these advantages and limitations in mind, we illustrate the power of EPO-GEMMs to uncover new biology. As one example, histopathological comparison of each tumor subtype revealed that late-stage *p53*-null CIN tumors, which are predominantly moderately differentiated, harbor undifferentiated regions that lack E-cadherin expression and resemble GS tumors produced by E-cadherin inactivation. These observations suggest that CIN and GS subtypes are subject to the forces of convergent evolution and, accordingly, we noted mutual exclusivity of *TP53* and *CDH1* mutations in primary gastric cancer from patients.

Other insights arising from our initial characterization of these models relate to metastatic organotropism. First, a subset of EPO-GEMM animals develop metastatic spread to the ovaries, an enigmatic but clinically important facet of gastric cancer presentation that has not been previously modeled. Our results demonstrate that these tumors can arise from different gastric cancer genotypes and that hematogenous migration is a viable route for their establishment. Second, EPO-GEMM models display genotype-specific patterns of metastatic organotropism that, though not previously known, were mirrored in patients. Hence, *Apc*-null GS tumors preferentially metastasized to the liver (a pattern that may extend to other cancer types^19,42^). *Msh2*-deficient MSI tumors were poorly metastatic, especially to lungs. *p53* inactivation conferred enhanced potential for peritoneal metastasis, mirroring a patient study in which alterations in the *TP53* pathway were among the most common mutations in diffuse gastric cancer with peritoneal metastases^55^. Finally, by targeting different recipient strains, we identified genotype-specific mechanisms of metastasis immune surveillance. While NK cells suppressed metastatic spread in all nonviral molecular subtypes of gastric cancer^47,48,56,57^, CD8^+^ T cells provided additional suppression of MSI tumors. This added layer of protection may explain the improved prognosis of MSI patients with gastric and other gastrointestinal cancers^58,59^.

Metastatic gastric cancer is a global health problem. Given the flexibility and breadth of gastric cancer EPO-GEMMs, these models may have a profound impact on gastric cancer research, analogous to the impact of the ‘KPC’ mouse on pancreatic cancer research^60^, and also enabling the study of a broad range of disease subtypes in reduced time and with less animal waste. Furthermore, as shown here, gastric cancer EPO-GEMMs allow for straightforward molecular studies on tumor–host interactions, now appreciated as central to cancer biology and therapy response. Future work will leverage single-cell approaches to characterize the extent of intratumoral heterogeneity in these models, which is a major challenge in the clinic. We anticipate that this platform will facilitate basic discovery efforts and accelerate the development of urgently needed therapeutic strategies for this deadly but understudied disease.

## Methods

### Ethical regulations

The research performed in this study complies with all relevant ethical regulations.

All mouse experiments were approved by the Memorial Sloan Kettering Cancer Center (MSKCC) Internal Animal Care and Use Committee (IACUC). All relevant animal use guidelines and ethical regulations were followed.

### Cell lines and compounds

The following cell lines were used in this study: *MP*, *MApc* and *MP.MSH*, which were derived from EPO-GEMM gastric tumors with these genotypes. To generate these cell lines, gastric tumors were minced, digested in DMEM containing 3 mg ml^−1^ dispase II (Gibco) and 1 mg ml^−1^ collagenase IV (C5138; Sigma) for 30 min at 37 °C, and plated on 10-cm culture dishes coated with 100 µg ml^−1^ collagen (PureCol; 5005; Advanced Biomatrix). Primary cultures were passaged at least three times to remove fibroblast contamination. Cells were maintained in a humidified incubator at 37 C with 5% CO_2_ and grown in DMEM supplemented with 10% FBS and 100 IU ml^−1^ penicillin/streptomycin. All cell lines used were tested and found negative for *Mycoplasma*.

### Reagents

For in vivo experiments, mice were treated with anti-CTLA-4 (200 µg; Bio X Cell; BE0131) or anti-PD-1 antibody (200 μg; RMP1-14, Bio X Cell; BE0146) three times per week via intraperitoneal injection. Anti-NK1.1 (250 µg; Bio X Cell; BE0036), anti-CD8 (200 µg; Bio X Cell; BE0061), anti-CD4 (200 µg; Bio X Cell; BP00031) or the respective isotype control (Bio X Cell; BE0290 or BE0090) was given twice per week by intraperitoneal injection.

### Animal studies

All mouse experiments were approved by the MSKCC IACUC. All relevant animal use guidelines and ethical regulations were followed. Mice were maintained under specific-pathogen-free conditions and food and water were provided ad libitum. Housing was on a 12-h light–dark cycle under standard temperature and humidity of approximately 18–24 °C and 40–60%, respectively. Survival data reflect the use of humane end points based on tumor burden and overall animal health. The following mice were used: C57BL/6N, Tg(Krt8-cre/ERT2)17Blpn/J (CK8-CreERT2, JAX stock 017947), B6;129-Gt(ROSA)26Sortm1(CAG-cas9*,-EGFP)Fezh/J (LSL-Cas9, JAX stock 024857), B6.FVB-Tg(Atp4b-cre)1Jig/JcmiJ (Atp4b-Cre, JAX 030656), *Nu*/*Nu* Nude mice (JAX stock 002019) and B6;129-Rag2tm1FwaII2rgtm1Rsky/DwlHsd (R2G2, purchased from Envigo). Mice were used at 8–12 weeks of age and kept in group housing. To induce gene recombination in CreERT2 mice, tamoxifen (0.5 mg per mouse), dissolved in corn oil, was administered via oral gavage for 4 consecutive days. Mice were randomly assigned to the experimental groups. While our experiments included both female and male mice, the cohorts were insufficiently powered to determine whether there were meaningful sex-based differences. Therefore, no a priori sex-based analyses were performed.

### EPO-GEMMs

The 8–12-week-old wild-type C57BL/6N mice were starved for 2 h before the procedure. Mice were anesthetized with isoflurane. Hair was removed from the surgical site (epigastrium), then the site was scrubbed with a povidone-iodine scrub (Betadine) and rinsed with 70% alcohol. After opening the peritoneal cavity, the stomach was mobilized and opened in the area of the forestomach (Extended Data Fig. 1a–d). Next, the inside of the stomach was rinsed with saline to remove any residual food (Extended Data Fig. 1e). Subsequently, the corpus/antrum region was used as a landmark, and the plasmid mix (diluted in 50 µl of elution buffer; QIAGEN) was injected via a 30-gauge syringe into the epithelial compartment in the corpus region generating a small depot (Extended Data Fig. 1f). Tweezer electrodes (NepaGene CUY650P3) were tightly placed around the injection bubble and an in vivo electroporator (NepaGene NEPA21 Type II Electroporator) was used to deliver two pulses of electrical current (75 V) given for 35-ms lengths at a 500-ms interval (Extended Data Fig. 1g). Immediately after electroporation, all the layers of the stomach were sutured at once in a continuous seam with absorbable sutures (Ethicon, VICRYL, 5-0, J493G), and the peritoneal cavity was rinsed with 0.5 ml prewarmed saline (Extended Data Fig. 1h). The peritoneal cavity was sutured (absorbable sutures, Ethicon, VICRYL, 5-0, J493G) and the skin closed with skin staples (Extended Data Fig. 1i). The mice were kept at 37 °C until awake. Post-surgery pain was managed with injections of buprenorphine (Buprenex, 0.5 mg kg^−1^; Covetrus) or meloxicam (2 mg kg^−1^; Covetrus) for the following 3 d and mice received DietGel 76A until they reached their pre-surgery weight (usually 5–8 d).

Tumor formation was assessed by palpation and ultrasound imaging; tumors could be detected when they reached a diameter of 3–4 mm. Mice were killed following early tumor development or at the end point (per IACUC guidelines, 1 cm^3^ tumor size and/or compromised health; tumor size was not exceeded). Genome editing in EPO-GEMM tumors was confirmed by Sanger sequencing.

To generate EPO-GEMM tumors in C57BL/6 wild-type mice, the following vectors and concentrations were used: a pT3-MYC transposon vector (5 μg), a Sleeping Beauty transposase (SB13; 1 μg) and/or a pX330 CRISPR/Cas9 vector (20 μg; Addgene #42230) targeting the respective tumor-suppressor genes. The Sleeping Beauty transposase (SB13) and the pT3 transposon vector were a generous gift from X. Chen (University of California, San Francisco). The pX330 vector (Addgene plasmid #42230) was a gift from F. Zhang of the Broad Institute.

The following sgRNAs were used to target the respective tumor-suppressor gene locus:

- p53: ACCCTGTCACCGAGACCCC
- APC: GCAGGAACCTCATCAAAACG
- CDH1: CCCGTTGGCGTTTTCATCAT
- MSH2: GACAAAGATTGGTTAACCAG
- PTEN: GTTTGTGGTCTGCCAGCTAA

To generate the pX330 vector containing two sgRNAs, the vector was opened using the *XbaI* cloning site and the sgRNA-cassette containing the second guide was PCR cloned into the vector using the following primers: XbaI U6 forward: ATGCTTCTAGAGAGGGCCTATTTCCCATGATT and NheI gRNA scaffold reverse: ATGTCGCTAGCTCTAGCTCTAAAACAAAAAAGC.

To generate EPO-GEMM tumors in CK8-CreERT2;LSL-Cas9 or Atp4b-Cre;LSL-Cas9 mice, the following vectors and concentrations were used: a pT3-MYC transposon vector (10 μg), a pT3-U6 transposon vector (20 μg) targeting the respective tumor-suppressor genes and a Sleeping Beauty transposase (SB13; 6 μg). The pT3-MYC transposon vector (Addgene #92046) was a generous gift from X. Chen. The sgRNAs used to target the respective tumor-suppressor gene loci were the same as outlined above.

### Statistics and reproducibility

Statistical analysis was performed as described in detail in the figure legends.

No data or animals were excluded from the analyses. The investigators were not blinded to allocation during experiments and outcome assessment. No statistical methods were used to predetermine sample sizes but our sample sizes are similar to those reported in previous publications^19,21^. Data distribution was assumed to be normal but this was not formally tested. For in vivo experiments mice were randomly assigned to the treatment groups after a tumor was detectable by abdominal palpation or ultrasound imaging. In vitro samples were randomly assigned to the respective groups. Further information on research design is available in the Nature Research Reporting Summary linked to this article.

### In vivo immune-checkpoint blockade

Tumors were initiated by in vivo electroporation as outlined above. After tumors reached a diameter of 4–5 mm as confirmed by abdominal palpation and ultrasound imaging, mice were randomly assigned to treatment with either anti-CTLA-4 (clone 9H10, cat. no. BE0131), anti-PD-1 (clone RMP1-14, Bio X Cell, cat. no. BE0146) or IgG control (Bio X Cell, cat. no. BE0087) and survival was monitored afterwards. Animals were killed when they reached humane end points as defined by the IACUC at MSKCC (1 cm^3^ tumor size and/or compromised health).

### Experimental metastasis assays

For tail vein injections, 1 × 10^5^ *MP*, *MPMsh2* or *MApc* gastric tumor cells were resuspended in 400 μl PBS and injected into 8–12-week-old C57BL/6N, Nude or R2G2 mice. For intrasplenic injections, 1 × 10^5^ *MApc* gastric tumor cells were resuspended in 50 μl DMEM and injected into the spleen, followed by surgical removal of the splenic half harboring the injection site (hemi-splenectomy), to avoid tumor formation in the spleen as a confounder while preserving splenic function.

### Analysis of metastasis burden

The presence of metastases in various organs was determined at experimental endpoint by gross examination under a dissecting scope. For liver and lung metastasis, metastatic burden was further quantified from single hematoxylin and eosin (H&E)-stained sections cut across the largest organ plane by counting individual lesions per section or estimating percentage of tumor area per organ slice.

### Histological analysis

Tissues were fixed overnight in 10% formalin, embedded in paraffin and cut into 5-μm sections. Sections were subjected to H&E staining. Immunohistochemical and immunofluorescence staining was performed following standard protocols. The following primary antibodies were used: E-cadherin (1:500 dilution, BD Bioscience, 610181), H+K (1:1,000 dilution, MBL International Corporation, D032-3), Ki67 (1:100 dilution, Abcam, AB16667), CK8 (1:1,000 dilution, BioLegend, 904801), MSH2 (1:200 dilution, Cell Signaling, D24B5), MYC (1:100 dilution, Abcam, AB32072), vimentin (1:200 dilution, Cell Signaling, 5741), B-catenin (1:200 dilution, BD Bioscience, 610153), MUC6 (1:100 dilution, LsBio, LS-C312108-0.1), CD45 (1:100 dilution, Cell Signaling, 70257), CD3 (1:100 dilution, Abcam, ab5690) and CD8 (1:2,000 dilution, Abcam, ab217344).

### Flow cytometry

For in vivo sample preparation, gastric tumors were processed into small pieces, digested in RPMI containing 2 mg ml^−1^ collagenase D and 100 µg ml^−1^ DNase I for 30 min at 37 °C, filtered through a 70-μm strainer and washed with PBS. Red blood cell lysis was achieved with an ACK (ammonium-chloride-potassium) lysis buffer (Lonza). Cells were washed with PBS, resuspended in FACS buffer and used for subsequent analysis. The following fluorophore-conjugated antibodies were used (‘m’ prefix denotes anti-mouse): m.CD45 (AF700, 1:200 dilution, BioLegend cat. no. 103128, lot B295205), m.CD3 (PE-Cy7, 1:100 dilution, BioLegend, cat. no. 100220, lot B284568), CD3 (AF488, 1:100 dilution, BioLegend, cat. no. 100210, lot B284975), CD4 (BUV395, 1:50 dilution, BD, cat. no. 563790, lot 9275330), CD8 (PE-Cy7, 1:50 dilution, BioLegend, cat. no. 100722, lot B282418), CD11c (BV650, 1:200 dilution, BioLegend, cat. no. 117339, lot B296085). m.CD3 (BV650, 1:300 dilution, BioLegend, cat. no. 100229), m.CD4 (BUV737, 1:200 dilution, BD, cat. no. 564298), m.CD8 (FITC, 1:300 dilution, BioLegend, cat. no. 100706) and m.CDllc (BV785, 1:200 dilution, BioLegend, cat. no. 117335). Flow cytometry was performed on a LSRFortessa instrument (BD Biosciences) using FACSDiva (v.8.0, BD Biosciences) software and data were analyzed using FlowJo (v.10.1, TreeStar).

### RNA extraction, RNA-seq library preparation and sequencing

Total RNA was isolated from MP, MP.MSH2 and MAPC tumors or healthy stomach (from untreated mice). Library preparation and sequencing were performed at the Integrated Genomics Operation Core at MSKCC. RNA-seq libraries were prepared from total RNA. After RiboGreen quantification and quality control by Agilent BioAnalyzer, 100–500 ng of total RNA underwent polyA selection and TruSeq library preparation according to instructions provided by Illumina (TruSeq Stranded mRNA LT kit, RS-122-2102), with eight cycles of PCR. Samples were barcoded and run on a HiSeq 4000 or HiSeq 2500 in a 50-bp/50-bp paired end run, using the HiSeq 3000/4000 SBS kit or TruSeq SBS kit v.4 (Illumina).

### RNA-seq read mapping, differential gene expression analysis and heat map visualization

RNA-seq data were analyzed by removing adaptor sequences using Trimmomatic^61^. RNA-seq reads were then aligned to GRCm38.91 (mm10) with STAR^62^ and transcript count was quantified using featureCounts^63^ to generate a raw count matrix. Analysis of differential gene expression between experimental conditions (using more than two independent biological replicates per condition) and adjustment for multiple comparisons were performed using DESeq2 package^64^, implemented in R (http://cran.r-project.org/). DEGs were defined as those with >twofold change in gene expression with adjusted *P* value < 0.05. For heat map visualization of DEGs, samples were *z* score-normalized and plotted using pheatmap package in R.

### Functional annotation of gene sets

Pathway enrichment analysis was performed in the resulting gene clusters (Fig. 4a) with the Reactome database using enrichR^65^. Significance of the tests was assessed using combined score, described as *c* = log(*P*) × z, where *c* is the combined score, *P* is Fisher’s exact test *P* value, and z is *z* score for deviation from expected rank.

### Gene set enrichment analysis

Gene set enrichment analysis (GSEA)^66^ was performed using the GSEAPreranked tool for conducting GSEA of data derived from RNA-seq experiments (v.2.07) against signatures in the MSigDB database (http://software.broadinstitute.org/gsea/msigdb). The metric scores were calculated using the sign of the fold change multiplied by the inverse of the *P* value.

### Gene signature score and immune cell type abundance estimation

A rank-based single-sample gene-set scoring method was calculated using package singscore in R^67^. Immune cell abundance estimation was based on the LM22 signature^40^, which contains a 547-gene signature matrix from 22 human immune cell types. LM22 signature and singscore were used to estimate gene expression profiles for each LM22 cell type.

### CNA analysis

CNAs were inferred from sparse whole-genome sequencing data as described previously^68,69^. In brief, 1 μg of bulk genomic DNA was extracted from gastric tumors using the DNeasy Blood and Tissue kit (QIAGEN) and sonicated using the Covaris instrument. Sonicated DNA was subsequently end-repaired/A-tailed, followed by ligation of TruSeq dual indexed adaptors. Indexed libraries were enriched via PCR and sequenced in multiplex fashion using the Illumina HiSeq 2500 Instrument to achieve roughly 1 × 10^6^ uniquely mappable reads per sample, a read count sufficient to allow copy-number inference to a resolution of approximately 400 kb. For data analysis, uniquely mapped reads were counted in genomic bins corrected for mappability. Read counts were subsequently corrected for guanine/cytosine content, normalized and segmented using circular binary segmentation. Segmented copy-number calls were illustrated as relative gains and losses to the median copy number of the entire genome. Broad events (chromosome-wide and several megabase-sized events) are discernible in a genome-wide manner.

### Whole-exome sequencing

A total of 1 μg bulk genomic DNA was extracted from gastric tumors using the DNeasy Blood and Tissue kit (QIAGEN) and WES was conducted and sequenced by BGI. The data were then processed through the Illumina (HiSeq) Exome Variant Detection Pipeline for detecting variants by the Bioinformatics Core at MSKCC. First, the FASTQ files were processed to remove any adaptor sequences at the end of the reads using cutadapt (v.1.6). The files were then mapped using the BWA mapper (bwa mem v.0.7.12). After mapping, the SAM files were sorted and read group tags added using the PICARD tools. After sorting in coordinate order, the BAMs were processed with PICARD MarkDuplicates. The marked BAM files were then processed using the GATK toolkit (v.3.2) according to the best practices for tumor–normal pairs. They were first realigned using ABRA (v.0.92) and then the base quality values were recalibrated with the BaseQRecalibrator. Somatic variants were then called in the processed BAMs using muTect (v.1.1.7) for SNV and the Haplotype caller from GATK with a custom post-processing script to call somatic indels. Based on the information provided by Agilent SureSelect XT Mouse All Exon kit, the total exome coverage was ~49.6 MB. This coverage length was used to calculate mutations per MB, and these values were compared to publicly available mutational data downloaded from elsewhere^30^.

### Human clinical data analysis

For transcriptomic analysis, TCGA stomach adenocarcinoma RNA-seq data were downloaded through the R package TCGAbiolinks^70^ to retrieve molecular subtypes and the raw and normalized (TPM) count tables. Patients with matched healthy and tumor samples were identified and used to run subtype-specific differential expression analysis. Results were used to calculate the rank score for GSEA and compared to GSEA results from the EPO-GEMMs. Microarray data from GSE62254 were downloaded and processed through R package limma^71^. DEGs between different molecular subtypes were identified and used for GSEA. Normalized enrichment scores (NES) were plotted and compared to EPO-GEMMs.

For metastasis analysis, human datasets were obtained through either the MSKCC Clinical Sequencing Cohort (MSK-IMPACT) via cBioPortal^72,73^ or the MSK-MET cohort^42^, as indicated in the text. Metastatic samples were clinically annotated and their genetic alterations were assessed by IMPACT sequencing. For the liver/lung/peritoneum tropism analysis (Fig. 5o–q), MSK-IMPACT samples were selected as follows: (1) cancer type, esophagogastric cancer; (2) sample type, metastasis; and (3) genotype (MUT: APC, MUT: TP53 or MSI_TYPE: instable). Then, the fraction of selected samples that was located in the liver, lung or peritoneum was calculated as a percentage of all metastatic sites. For the MSS versus MSI metastasis analysis (Fig. 7f), MSK-IMPACT samples were selected as follows: (1) cancer type: esophagogastric cancer; (2) MSI_TYPE: stable or instable. Then, the fraction of selected samples that were derived from metastatic sites was calculated as percentage of all (primary + metastatic) samples. For the liver/lung metastasis incidence analysis from the MSK-MET cohort (Extended Data Fig. 8d), patients with stomach adenocarcinoma were filtered by the presence of WNT pathway or *TP53* mutations, and then analyzed for the incidence of liver or lung metastases, as described in the published study^42^. Statistical comparisons were performed through contingency table analyses using Fisher’s exact test in Prism v.7.0 (GraphPad Software) for the MSK-IMPACT cohort, or as described previously for the MSK-MET cohort^42^. Sex- or gender-based analyses were not planned a priori; thus, sex or gender were not considered in the human data analysis.

### Immunoblotting

Cell lysis was performed using RIPA buffer (Cell Signaling Technology) supplemented with phosphatase inhibitors (5 mmol l^−1^ sodium fluoride, 1 mmol l^−1^ sodium orthovanadate, 1 mmol l^−1^ sodium pyrophosphate and 1 mmol ^−1^ β-glycerophosphate) and protease inhibitors (Protease Inhibitor Cocktail Tablets, Roche). Protein concentration was determined using a Bradford Protein Assay kit (Bio-Rad). Proteins were separated by SDS–PAGE and transferred to polyvinyl difluoride membranes (Millipore) according to the standard protocols. Membranes were immunoblotted overnight at 4 °C with antibodies against MSH2 (Cell Signaling, D24B5) or β-actin (Cell Signaling, 4970) in 5% BSA in TBS-blocking buffer. Membranes were incubated with secondary anti-rabbit antibody (Cell Signaling, 7074) for 1 h at room temperature. Blots were developed in PerkinElmer’s Western Lightning ECL as per the manufacturer’s instructions.

### Statistical analysis and figure preparation

Data are presented as mean ± s.e.m. Statistical analysis was performed by Student’s *t*-test, ANOVA, Mann–Whitney *U*-test, Wilcoxon signed-rank test or Fisher’s exact test using Prism v.6.0 or 7.0 (GraphPad Software), as indicated in the respective figure legends. *P* values < 0.05 were considered statistically significant. Excel (Microsoft) was used to calculate survival. Subsequently, survival was determined using the Kaplan–Meier method, with a log-rank test used to determine statistical significance. No statistical method was used to predetermine sample size in animal studies. Animals were allocated at random to treatment groups. Figures were prepared using BioRender for scientific illustrations and Illustrator CC 2021 (Adobe).

### Reporting summary

Further information on research design is available in the Nature Portfolio Reporting Summary linked to this article.

### Supplementary information

Reporting Summary

Supplementary Table 1 Supplementary Tables 1–21.

### Source data

Source Data Figs. 1–7 and Extended Data Figs. 1–9 Statistical source data for Figs. 1–7 and Extended Data Figs. 1–9.

Source Data Extended Data Fig. 6 Unprocessed western blot for Extended Data Fig. 6.

## Figures and Tables

**Extended Data Fig. 10:**
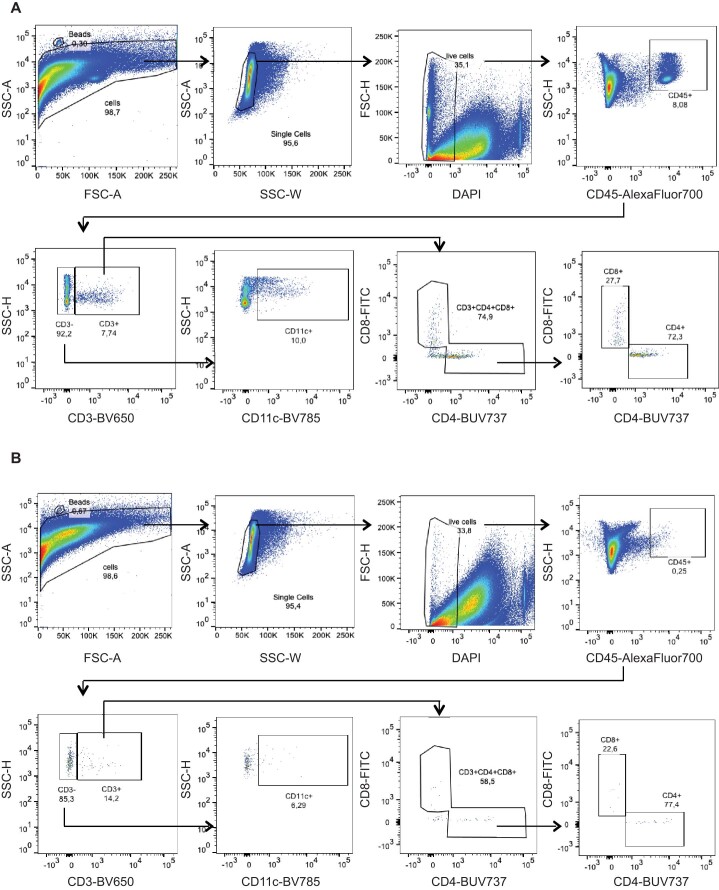
Flow cytometry gating strategy. (**a, b**) Representative flow cytometric analysis of MSI gastric tumors after treatment with antibodies targeting CTLA-4 (A) or IgG control (B). Placement of gates was based on FMO controls.

## Data Availability

RNA-seq, WES and Sparse whole-genome sequencing data that support the findings of this study have been deposited in the Gene Expression Omnibus under accession codes GSE199261, PRJNA1013074 and PRJNA818675. Datasets derived from this resource that support the findings of the present study are available in Supplementary Tables 1–21. The human gastric cancer genomic data were derived from the TCGA Research Network at http://cancergenome.nih.gov/. The dataset derived from this resource that supports the findings of this study is available at https://gdc.cancer.gov/about-data/publications/pancanatlas. All other data supporting the findings of this study are available from the corresponding authors on reasonable request.
