# Supplementary material for: Somatic mouse models of gastric cancer reveal genotype-specific features of metastatic disease
Source: Nat Cancer. 2024 Jan 4;5(2):315–29. doi: 10.1038/s43018-023-00686-w (PMC10899107; doi:10.1038/s43018-023-00686-w)
Supplement: Supplementary file 1 — Reporting Summary [file 43018_2023_686_MOESM1_ESM.pdf]

nature portfolio

Corresponding author(s): Dr. Scott Lowe, Dr. Josef Leibold

Last updated by author(s): Sep 10, 2023

Reporting Summary

Nature Portfolio wishes to improve the reproducibility of the work that we publish. This form provides structure for consistency and transparency in reporting. For further information on Nature Portfolio policies, see our Editorial Policies and the Editorial Policy Checklist.

Statistics

For all statistical analyses, confirm that the following items are present in the figure legend, table legend, main text, or Methods section.

n/a	Confirmed	
☐	☒	The exact sample size (n) for each experimental group/condition, given as a discrete number and unit of measurement
☐	☒	A statement on whether measurements were taken from distinct samples or whether the same sample was measured repeatedly
☐	☒	The statistical test(s) used AND whether they are one- or two-sided<br>*Only common tests should be described solely by name; describe more complex techniques in the Methods section.*
☒	☐	A description of all covariates tested
☐	☒	A description of any assumptions or corrections, such as tests of normality and adjustment for multiple comparisons
☐	☒	A full description of the statistical parameters including central tendency (e.g. means) or other basic estimates (e.g. regression coefficient) AND variation (e.g. standard deviation) or associated estimates of uncertainty (e.g. confidence intervals)
☐	☒	For null hypothesis testing, the test statistic (e.g. F, t, r) with confidence intervals, effect sizes, degrees of freedom and P value noted<br>*Give P values as exact values whenever suitable.*
☒	☐	For Bayesian analysis, information on the choice of priors and Markov chain Monte Carlo settings
☒	☐	For hierarchical and complex designs, identification of the appropriate level for tests and full reporting of outcomes
☒	☐	Estimates of effect sizes (e.g. Cohen's d, Pearson's r), indicating how they were calculated

Our web collection on statistics for biologists contains articles on many of the points above.

Software and code

Policy information about availability of computer code

Data collection	LSR-Fortessa, Cytek Aurora (CYTEK), Microsoft Excel for Mac 2011
Data analysis	FlowJo 10.1, GraphPad Prism V6 and V7, Adobe Illustrator version 25.2.3, lmageJ, RNAseq analysis was performed with the following software: HTseq v0.5.3, picard tools vl.124, R v3.2.0, STAR v2.5.0a, samtools v0.1.19, Microsoft Excel for Mac 2011, FACS Diva version 8.0, Trimmomatic version 0.36

For manuscripts utilizing custom algorithms or software that are central to the research but not yet described in published literature, software must be made available to editors and reviewers. We strongly encourage code deposition in a community repository (e.g. GitHub). See the Nature Portfolio guidelines for submitting code & software for further information.

Data

Policy information about availability of data

All manuscripts must include a data availability statement. This statement should provide the following information, where applicable:

- Accession codes, unique identifiers, or web links for publicly available datasets
- A description of any restrictions on data availability
- For clinical datasets or third party data, please ensure that the statement adheres to our policy

RNA-Seq, WES and Sparse whole-genome sequencing data that support the findings of this study have been deposited in the Gene Expression Omnibus (GEO) under accession codes GSE199261, PRJNA1013074, and PRJNA818675. The human gastric cancer genomic data were derived from the TCGA Research Network: http://

cancergenome.nih.gov/. The data-set derived from this resource that supports the findings of this study is available under https://gdc.cancer.gov/about-data/publications/pancanatlas. Source data for Figs. 4A-B, Fig. 4D and Extended Data Fig. 6A, Extended Data Figs. 7A-B and Extended Data Fig. 7G have been provided as Source Data files. All other data supporting the findings of this study are available from the corresponding author on reasonable request.

Research involving human participants, their data, or biological material

Policy information about studies with human participants or human data. See also policy information about sex, gender (identity/presentation), and sexual orientation and race, ethnicity and racism.

Reporting on sex and gender	No a priori sex- or gender-based analyses were performed on the human data, so we refrain from drawing conclusions regarding sex/gender-based differences
Reporting on race, ethnicity, or other socially relevant groupings	Race, ethnicity, or other socially relevant groupings were not considered or analyzed in this study.
Population characteristics	Human gastric cancer genomic data (Fig. 1B) were derived from the TCGA Research Network: http://cancergenome.nih.gov/. Human gastric cancer transcriptional data (Fig. 4B, Extended Data Figure 7C-E, G) were derived from DOI: 10.1038/nature13480. For analysis of the incidence of liver, lung and peritoneal metastases in human gastric cancer patients (Fig. 5 Q-S, Extended Data Figure 8D) public datasets available under DOI: 10.1016/j.cell.2022.01.003 were used.
Recruitment	not applicable
Ethics oversight	not applicable

Note that full information on the approval of the study protocol must also be provided in the manuscript.

Field-specific reporting

Please select the one below that is the best fit for your research. If you are not sure, read the appropriate sections before making your selection.

☒ Life sciences ☐ Behavioural & social sciences ☐ Ecological, evolutionary & environmental sciences

For a reference copy of the document with all sections, see nature.com/documents/nr-reporting-summary-flat.pdf

Life sciences study design

All studies must disclose on these points even when the disclosure is negative.

Sample size	no statistical methods were used to pre-determine sample size. Sample sizees were estimated based on preliminary experiments, with an effort to achieve a minimum of n=5 mice per treatment group
Data exclusions	no data was excluded
Replication	All experiments were repeated in 3 independent replicates and/or from at least 3 different subjects in independent experiments. All attempts at replication were successful. Exceptions apply to proof-of-concept experiments presented in Extended Data Figures 2N-T and 5K-N as indicated in the respective figure legends.
Randomization	Mice were randomly assigned to treatment groups before electroporation or after palpable tumors had formed.
Blinding	Mouse conditions were observed by an operator who was blinded to the treatment groups in addition to the main investigator who was not blind to treatment allocation. Analysis of data was not perfomred in blinded fashion. Data analysis are based on objective measurable data (tumor burden, number of metastases).

Reporting for specific materials, systems and methods

We require information from authors about some types of materials, experimental systems and methods used in many studies. Here, indicate whether each material, system or method listed is relevant to your study. If you are not sure if a list item applies to your research, read the appropriate section before selecting a response.

Materials & experimental systems

n/a	Involved in the study
☐	☒ Antibodies
☐	☒ Eukaryotic cell lines
☒	☐ Palaeontology and archaeology
☐	☒ Animals and other organisms
☒	☐ Clinical data
☒	☐ Dual use research of concern
☒	☐ Plants

Methods

n/a	Involved in the study
☒	☐ ChIP-seq
☐	☒ Flow cytometry
☒	☐ MRI-based neuroimaging

Antibodies

Antibodies used

the following antibodies were used for flowcytometry:
('m' prefix denotes anti-mouse): m.CD45 (AF700, 1:200, Biolegend Cat.# 103128, Lot# B295205), m.CD3 (PE-Cy7, 1:100, Biolegend, Cat.# 100220, Lot# B284568), CD3 (AF488, 1:100, Biolgened, Cat.# 100210, Lot# B284975), CD4 (BUV395, 1:50, BD, Cat.# 563790, Lot# 9275330), CD8 (PECy7, 1:50, Biolegend, Cat.# 100722, Lot# B282418), CD11c (BV650, 1:200, Biolegend, Cat.# 117339, Lot# B296085), m.CD3 (BV650, 1:300, Biolegend, #100229), m.CD4 (BUV737, 1:200, BD, #564298), m.CD8 (FITC, 1:300, Biolgend, #100706), m.CDllc (BV785, 1:200, Biolegend, #117335).

m.CD45 (AF700, 1:200, Biolegend Cat.# 103128, Lot# B295205), m.CD3 (PE-Cy7, 1:100, Biolegend, Cat.# 100220, Lot# B284568), CD3 (AF488, 1:100, Biolgened, Cat.# 100210, Lot# B284975), CD4 (BUV395, 1:50, BD, Cat.# 563790, Lot# 9275330), CD8 (PECy7, 1:50, Biolegend, Cat.# 100722, Lot# B282418), CD11c (BV650, 1:200, Biolegend, Cat.# 117339, Lot# B296085).

The follwoing antibodies were used for immunehistochemistry:
E-Cadherin (1:500,BD Bioscience, 610181), H+K (1:1000, MBL International Corporration, D032-3), Ki67 (1:100, Abcam, AB16667), CK8 (1:1000, BioLegend, 904801), MSH2 (1:200, Cell Signaling, D24B5), MYC (1:100, Abcam, AB32072), Vimentin (1:200, Cell Signaling, 5741), anti MUC6 (1:100, LsBio, LS-C312108-0.1), B-Catenin (1:200, BD Bioscience, 610153), CD8 (1:2000, abcam, ab217344)

The follwoing antibodies were used for immunofluorescence staining:
CD45 (1:100, Cell Signaling, 70257), CD3 (1:100, abcam, ab5690)

Validation

All used antibodies were titrated. All the antibodies are validated for use in flow cytometry or immunhistochemistry or immunofluorescence. Data are available on the manufacturer's website the following antobodies have been validated by the manufacturer:

Flowcytometry:
https://www.biolegend.com/en-us/products/alexa-fluor-700-anti-mouse-cd45-antibody-3407
https://www.biolegend.com/ja-jp/products/pe-cyanine7-anti-mouse-cd3-antibody-6060
https://www.biolegend.com/en-us/products/alexa-fluor-488-anti-mouse-cd3-antibody-2835?GroupID=BLG242
https://www.bdbiosciences.com/en-br/products/reagents/flow-cytometry-reagents/research-reagents/single-color-antibodies-ruo/buv395-rat-anti-mouse-cd4.563790
https://www.biolegend.com/de-at/products/pe-cyanine7-anti-mouse-cd8a-antibody-1906
https://www.biolegend.com/en-us/products/brilliant-violet-650-anti-mouse-cd11c-antibody-8840?GroupID=BLG11937
https://www.biolegend.com/de-at/products/brilliant-violet-650-anti-mouse-cd3-antibody-7843?GroupID=BLG242
https://www.bdbiosciences.com/en-us/products/reagents/flow-cytometry-reagents/research-reagents/single-color-antibodies-ruo/buv737-rat-anti-mouse-cd4.612761
https://www.biolegend.com/en-gb/products/fitc-anti-mouse-cd8a-antibody-153?GroupID=BLG2559
https://www.biolegend.com/de-de/soluble-mhc/brilliant-violet-785-anti-mouse-cd11c-antibody-7963

Immune histochemistry:
https://www.bdbiosciences.com/en-us/products/reagents/microscopy-imaging-reagents/immunofluorescence-reagents/purified-mouse-anti-e-cadherin.610182
https://www.mblbio.com/bio/g/dtl/A/index.html?pcd=D032-3
https://www.abcam.com/products/primary-antibodies/ki67-antibody-sp6-ab16667.html
https://www.biolegend.com/Files/Images/media_assets/pro_detail/datasheets/904801_V06.pdf
https://www.cellsignal.com/products/primary-antibodies/msh2-d24b5-xp-rabbit-mab/2017?_requestid=772840
https://www.abcam.com/products/primary-antibodies/c-myc-antibody-y69-chip-grade-ab32072.html
https://www.cellsignal.com/products/primary-antibodies/vimentin-d21h3-xp-rabbit-mab/5741?gclid=EAIaIQobChMI7ZGpifr-gAMVaZiDBx1nfQL6EAAYASAAEgKOaPD_BwE&gclsrc=aw.ds
https://www.lsbio.com/antibodies/muc6-antibody-muc-6-antibody-clone-clh5-ihc-ls-c312108/322074
https://www.bdbiosciences.com/en-au/products/reagents/microscopy-imaging-reagents/immunofluorescence-reagents/purified-mouse-anti-catenin.610153
https://www.abcam.com/products/primary-antibodies/cd8-alpha-antibody-epr21769-ab217344.html

Immunofluorescence staining:
https://www.cellsignal.com/products/primary-antibodies/cd45-d3f8q-rabbit-mab/70257?_requestid=347990
https://www.abcam.com/products/primary-antibodies/cd3-antibody-ab5690.html

Eukaryotic cell lines

Policy information about cell lines and Sex and Gender in Research

Cell line source(s)	cell lines were generated from the respective mouse tumor samples.
Authentication	the genetics of the cell lines was confirmed by sanger sequencing of the genetic locus targeted by the respective CRISPR/Cas9 single guide RNA.
Mycoplasma contamination	All cell lines were tested for mycoplasma and were found to be negative.
Commonly misidentified lines (See ICLAC register)	No commonly misidentified cell lines were used.

Animals and other research organisms

Policy information about studies involving animals; ARRIVE guidelines recommended for reporting animal research, and Sex and Gender in Research

Laboratory animals	C57BL/6N mice were males and females, 8-12- weeks old and obtained from the jackson Laboratory; Tg(Krt8-cre/ERT2)17Blpn/J (CK8-CreERT2, JAX stock #017947), B6;129-Gt(ROSA)26Sortm1(CAG-cas9*,-EGFP)Fezh/J (LSL-Cas9, JAX stock #024857), B6.FVB-Tg(Atp4b-cre)1Jig/JcmiJ (Atp4b-Cre, JAX #030656) and Nu/Nu Nude mice (JAX stock #002019) were obtained from jackson Laboratory (male and female), and B6;129-Rag2tm1FwaII2rgtm1Rsky/DwlHsd (R2G2, purchased from Envigo) mice male, 8-12- weeks old were obtained from envigo;
Wild animals	This study did not involve wild animals
Reporting on sex	Our experiments included both female and male mice. However, the cohorts were insufficiently powered to determine if there were meaningful sex-based differences. No a priori sex-based analyses were performed.
Field-collected samples	not applicable to this study
Ethics oversight	Memorial Sloan Kettering Cancer Center (MSKCC) Internal Animal Care and Use Committee

Note that full information on the approval of the study protocol must also be provided in the manuscript.

Flow Cytometry

Plots

Confirm that:

☒ The axis labels state the marker and fluorochrome used (e.g. CD4-FITC).

☒ The axis scales are clearly visible. Include numbers along axes only for bottom left plot of group (a 'group' is an analysis of identical markers).

☒ All plots are contour plots with outliers or pseudocolor plots.

☒ A numerical value for number of cells or percentage (with statistics) is provided.

Methodology

Sample preparation	For in vivo sample preparation, gastric tumors were processed into small pieces, digested in RPMI containing 2 mg/ml collagenase D and 100 µg/ml DNase I for 30 minutes at 37C, filtered through a 70-µm strainer and washed with PBS, then red blood cell lysis was achieved with an ACK (Ammonium-Chloride-Potassium) lysis buffer (Lonza). Cells were washed with PBS, resuspended in FACS buffer, and used for subsequent analysis.
Instrument	Fortessa 3, BD<br>Cytek Aurora (CYTEK)
Software	Collection: FACS Diva<br>Analysis: FlowJo 10.1
Cell population abundance	Cell sorting was not performed in this study.
Gating strategy	The starting cell population was gated on a SSC-A/FSC-A plot. Positive/negative populations were determined based on FMO controls

☒ Tick this box to confirm that a figure exemplifying the gating strategy is provided in the Supplementary Information.
