# Supplementary material for: Somatic mouse models of gastric cancer reveal genotype-specific features of metastatic disease
Source: Nat Cancer. 2024 Jan 4;5(2):315–29. doi: 10.1038/s43018-023-00686-w (PMC10899107; doi:10.1038/s43018-023-00686-w)

Unprocessed western blot

Corresponding to Extended Data Figure 6A
(Areas highlighted in red are displayed in the paper)

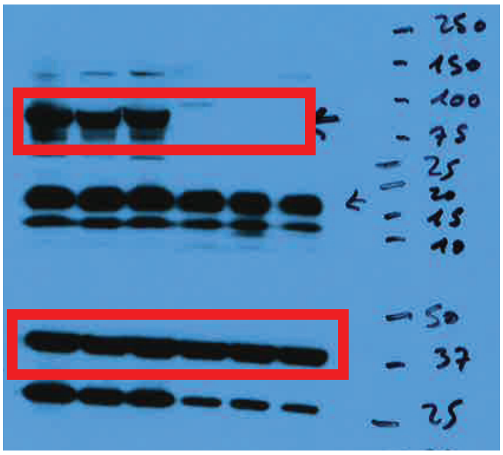

Supplement: Supplementary file 4 — Unprocessed western blot for Extended Data Fig. 6. [file 43018_2023_686_MOESM4_ESM.pdf]
